# Enteric dysfunction and enteropathogens among hospitalized south Asian and sub-Saharan African children

**DOI:** 10.1371/journal.pgph.0006873

**Published:** 2026-07-24

**Authors:** Abu Sadat Mohammad Sayeem Bin Shahid, Donna M. Denno, Kevin Kariuki, Doreen Rwigi, Amran Gazi, Lubaba Shahrin, Wilson Gumbi, James M. Njunge, Mohammod J. Chisti, Tahmeed Ahmed, Eric R. Houpt, Jie Liu, Mami Taniuchi, Benson O. Singa, Robert H. J. Bandsma, Wieger Voskuijl, Ali F. Saleem, Christina L. Lancioni, Ezekiel Mupere, Abdoulaye H. Diallo, Judd L. Walson, James A. Berkley, Kirkby D. Tickell

**Affiliations:** 1 The Childhood Acute Illness and Nutrition Network, Nairobi, Kenya; 2 Nutrition Research Division, International Centre for Diarrhoeal Disease Research Bangladesh (icddr, b), Dhaka, Bangladesh; 3 Department of Pediatrics, University of Washington, Seattle, Washington, United States of America; 4 Department of Global Health, University of Washington, Seattle, Washington, United States of America; 5 Centre for Microbiology Research, Kenya Medical Research Institute (KEMRI), Nairobi, Kenya; 6 Kemri-Welcome Trust Research Program, Kilifi, Kenya; 7 Division of Infectious Diseases and International Health, University of Virginia, Charlottesville, Virgina, United States of America; 8 School of Public Health, Qingdao University, Qingdao, China; 9 Departments of Medicine, Biomedical Engineering and Civil and Environmental Engineering, University of Virginia, Charlottesville, Virginia, United States of America; 10 Centre for Clinical Research, Kenya Medical Research Institute (KEMRI), Nairobi, Kenya; 11 Center for Global Child Health, Hospital for Sick Children, Toronto, Canada; 12 The Translational Medicine Program, Hospital for Sick Children, Toronto, Canada; 13 Amsterdam Institute for Global Child Health, Emma Children’s Hospital, Amsterdam, The Netherlands; 14 Paediatrics and Child Health, Aga Khan University, Karachi, Pakistan; 15 Department of Pediatrics, Oregon Health and Science University, Portland, Oregon, United States of America; 16 Department of Paediatrics and Child Health School of Medicine College of Health Sciences, Makerere University, Kampala, Uganda; 17 Department of Public Health, University Joseph Ki-Zerbo, Ouagadougou, Burkina Faso; 18 Departments of International Health, Medicine and Pediatrics, Johns Hopkins University, Baltimore, Maryland, United States of America; 19 Center for Tropical Medicine and Global Health, University of Oxford, Oxford, United Kingdom; North Carolina State University, UNITED STATES OF AMERICA

## Abstract

Enteropathogens and enteric dysfunction (ED) among acutely ill children in low-and-middle income countries (LMICs) may contribute to poor post-discharge outcomes. Enteropathogen prevalence and fecal ED biomarkers (myeloperoxidase, calprotectin, α-1-antitrypsin) from children aged 2–23 months hospitalized at nine LMIC facilities (n = 811) were compared to community children (n = 248). Host and pathogen correlates of ED biomarkers were identified through crude and adjusted linear mixed effect models, and Cox-proportional hazard models assessed ED biomarker associations with mortality in the 30-days following admission and 180-days following discharge. Invasive enteropathogens were more prevalent at admission (69%) than discharge (60%, p < 0.001) or among community children (61%, p = 0.004). Among the biomarkers, myeloperoxidase was higher at admission (2290 ng/ml, interquartile range [IQR]: 868, 6052, p = 0.014), and lower at discharge (1380 ng/ml, IQR: 611, 3193, p < 0.001) compared to community children (2268 ng/ml, IQR: 1051, 5421), while calprotectin was similar at admission (215 ug/ml, IQR: 77, 746, p = 0.351), but lower at discharge (170 ug/ml, IQR: 68, 369, p = 0.007) when compared the community children (252 ug/ml, IQR: 124, 691). α-1-antitrypsin concentrations were lower at admission (121 mg/l, IQR: 49, 303, p = 0.049) compared to the community (201 mg/ml, IQR: 100, 412), but more comparable at discharge (144 mg/ml, IQR: 69, 275, p = 0.380). Admission myeloperoxidase and calprotectin concentrations were associated with invasive enteropathogens detection (respectively, p = 0.003 and p = 0.002) and lower MUAC (respectively, p = 0.002 and p = 0.012), while admission α-1-antitrypsin was associated with breastfeeding, diarrhea, malaria and pneumonia (all p < 0.05). The only ED biomarker associated with mortality after confounder adjustment was fecal calprotectin (hazard ratio: 1.36, 95% CI:1.07,1.72, p = 0.011) at discharge. Enteric inflammation biomarker concentrations and enteropathogen prevalence were high at hospital admission, but by discharge were lower than among community peers. Interventions to prevent re-colonization with enteropathogens and increased enteric inflammation may improve child health in the post discharge period.

## Introduction

Pediatric mortality following discharge from hospitals in low- and middle-income countries (LMICs) is unacceptably high, with estimates suggesting that nearly half of deaths among hospitalized children occurring post-discharge [1–3]. Many of these post-discharge deaths occur within the first 45 days following hospitalization, and are attributable to broad range of acute and chronic conditions [3]. However, the risk of mortality remains elevated up to six or even 12 months after hospitalization suggesting that underlying vulnerabilities predispose these children to poor outcomes.

Enteric dysfunction (ED) due to various etiologies may contribute to poor outcomes after hospital discharge. Environmental enteric dysfunction (EED) is a prevalent, largely asymptomatic condition in LMICs characterized by small bowel injury, inflammation, increased gut permeability, translocation of microbes or microbial products, and systemic inflammation. EED is highly consequential to child growth and neurodevelopment, with impacts on health across the life course [4–6]. Frequent exposure to enteric pathogens has been associated with EED among children in the community [7–11]. Children admitted to hospital have a high prevalence of other conditions which can also cause ED, including malnutrition, acute gastrointestinal infections, and chronic infections such as HIV [5,12,13]. Furthermore, exposure to treatments, such as antibiotics and therapeutic foods, can disrupt the gut microbiome and intestinal homeostasis. Data from Zambia and Zimbabwe associated ED and systemic inflammation biomarkers with increased risk of mortality and rehospitalization in the post-discharge period among children with severe acute malnutrition [14]. Data from the cohort used for this analysis found detection lipopolysaccharide, a marker of translocation of gram negative bacteria or bacterial antigens, to be associated with mortality among hospitalized children [15]. A deeper understanding of ED, broadly defined and regardless of specific etiology, during acute illness may lead to interventions that prevent post-discharge morbidity and mortality.

Fecal myeloperoxidase and calprotectin are commonly used enteric inflammation biomarkers, while fecal α-1-antitrypsin is used to measure intestinal permeability [7,16]. These biomarkers have been associated with enteropathogens among community-based pediatric cohorts, particularly those classified as enteroinvasive [7]. However, few studies have examined ED biomarkers among acutely unwell children in LMICs. This analysis aimed to understand a) trends in ED biomarkers and enteropathogens in acutely unwell children across a nutritional spectrum in LMICs, b) enteropathogen, clinical, and sociodemographic correlates of ED, and c) ED biomarkers’ association with mortality in the 30-days following admission and 180-days following discharge.

## Methods

The Childhood Acute Illness and Nutrition (CHAIN) cohort characterized biomedical and social pathways to mortality among acutely ill young children [3]. Between November 2016 and January 2019, the CHAIN cohort enrolled 3,101 acutely ill children aged 2–23 months at nine hospitals: Dhaka and Matlab Hospitals (Bangladesh), Banfora Referral Hospital (Burkina Faso), Kilifi County, Mbagathi County and Migori County Hospitals (Kenya), Queen Elizabeth Hospital (Malawi), Karachi Civil Hospital (Pakistan), and Mulago National Referral Hospital (Uganda). These hospitals serve a range of urban and rural communities with varying healthcare access and disease endemicity, including HIV and malaria.

### Study design, setting and population

Enrollment was stratified by mid-upper-arm circumference (MUAC) to oversample undernourished children in a 2-1-2 ratio: no wasting (MUAC ≥12.5 cm [age ≥ 6 months] or MUAC≥12.0 cm [age < 6 months]), moderate wasting (MUAC 11.5–12.5 cm [age ≥ 6 months] or MUAC 11.0–12.0 cm [age < 6 months]), and severe wasting or kwashiorkor (MUAC <11.5 cm [age ≥ 6 months] or MUAC <11.0 cm [age < 6 months], or bilateral pedal edema) at hospital admission. To provide normative social and biological data for local populations, similarly aged community participants were recruited from households proximate to the index hospitalized child’s home using pseudo-random selection (3^rd^ house north of index home), if they had no hospital admission in the 14 days prior to contact with the study team and did not currently have an illness requiring medical attention.

Definitions, procedures, data, and sample collection and processing were harmonized across sites through training, standard operating procedures and case report forms (available at https://chainnetwork.org/resources/). Stool samples were collected at admission and discharge for hospitalized children and at a single timepoint for community participants.

The CHAIN nested case cohort (NCC) analyzed CHAIN samples to gain further insights into mortality, and was powered to detect a Hazard Ratio of 1.5 with 80% power in both the 30-day and 180-day mortality analyses.[17] Therefore, the CHAIN NCC randomly selected 24% of children in the CHAIN cohort, all remaining deaths, and 30 community children from each site (to provide reference values for biomarkers without well-established clinical cutoffs) to undergo a panel of analyses including fecal ED biomarker quantification by ELISA and TaqMan probe-based real-time PCR Arrays for detection of enteropathogens [17].

### Ethics statement

Formal written informed consent was obtained from parents or guardians of the children. Ethical approval was granted by the Oxford Tropical Research Ethics Committee, UK; the Kenya Medical Research Institute, Kenya; Makerere University School of Biomedical Sciences Research Ethics Committee and the Uganda National Council for Science and Technology, Uganda; Aga Khan University, Pakistan; International Centre for Diarrhoeal Disease Research, Bangladesh (icddr,b); The University of Malawi; The Centre Muraz, Burkina Faso; and the Hospital for Sick Children, Canada.

### Laboratory analysis and data preprocessing

Biological samples were stored at -80°C and shipped on dry ice. Stool myeloperoxidase, calprotectin, and α-1-antitrypsin were quantified by ELISA assay and absolute concentrations were calculated for 15mg of stool using manufacturer’s standard dose response curves at KEMRI’s Nairobi laboratory. For myeloperoxidase (Immunodiagnostik AG, Bensheim, Germany, intra-assay repeatability coefficient of variation (CV) 5%, inter-assay reproducibility CV 9–12%), [18] samples were diluted 1:10 and run on a final dilution of 1:500. α-1-antitrypsin (Immunodiagnostik AG, Bensheim, Germany, intra-assay repeatability CV 5–9%, inter-assay reproducibility CV 10–12%) [19] at a final dilution of 1:25000, while calprotectin (Immunodiagnostik AG, Bensheim, Germany, intra-assay repeatability CV 3–5%, inter-assay reproducibility CV 9–12%) [20] had a final dilution of 1:2500. Further dilutions were run if biomarker concentrations exceeded the standard curve range. Up to five samples per plate were duplicated to assess the coefficient of variance. All plates were read on ELx 808 ELISA plate reader (BioTek, USA) at wavelength of 450 nanometers and background absorbance was subtracted. Absolute quantification was performed against standard curves created using the manufacturer’s standards and compared against manufacturer provided controls.

TaqMan Array Cards (TAC) were run in Kenya and Bangladesh [17]. Total nucleic acid was extracted from rectal swabs using the QIAamp Fast DNA Stool Mini kit (Qiagen, Valencia, CA). 46 μl nucleic acid extract from rectal swab was mixed with AgPath One Step RT-PCR reagents (Thermo Fisher, Carlsbad, CA) in a 100 μl reaction, then loaded into the TAC card and run in a ViiA 7 or QuantStudio 7 Flex Real Time PCR system (Thermo Fisher, CA). TAC cards were customized to detect 29 pathogens commonly associated with diarrhea [S1 Appendix]. Cycle threshold (Ct) value of 30 was set as a threshold for analysis, whereby a Ct ≥ 30 was considered negative [21].

### Statistical analysis

Sociodemographic and clinical characteristics at hospital admission and discharge, and among the community participants, were presented using descriptive statistics. Enteropathogen prevalence adjusted for selection into the NCC at each timepoint was evaluated using chi-Square tests comparing admission to discharge, and each timepoint to the community. ED biomarker concentrations adjusted for selection into the NCC were described at admission, discharge, and for the community children using median (interquartile range [IQR]).Crude linear regression compared admission and discharge ED biomarker values to those from community children, rather than directly comparing the two hospitalized timepoints to avoid paired data. Sensitivity analyses stratifying hospitalized children into those with and without diarrhea reported as a presenting complaint were conducted. A final sensitivity analysis included only children with both admission and discharge samples to ensure trends between timepoints were not attributable to inpatient mortality.

### Correlates of ED biomarkers

Enteropathogens were grouped into categories, mirroring the approach taken by Kosek et al.[7] Dehydrating viruses that cause only limited mucosal disruption included adenovirus, astrovirus, norovirus and rotavirus. Invasive pathogens that cause mucosal disruption included *Aeromonas* spp*.*, *Campylobacter* spp., enteroaggregative *Escherichia coli* (EAEC), typical and atypical enteropathogenic *E. coli* (EPEC), *Salmonella* spp.*,* and *Shigella* spp. Enterotoxigenic *E. coli* (ETEC) was a third group as it causes secretory diarrhea with limited mucosal disturbances. Finally, *Giardia* spp. and *Cryptosporidium* spp. were modeled separately. Univariate linear mixed effect models with random effects for site estimated the association of age, anthropometry (nutritional edema, MUAC, length-for-age Z score [LAZ]), breastfeeding (exclusive/partial/none), clinical conditions (malaria, diarrhea, pneumonia, sepsis), and enteropathogen detection (dehydrating, enteroinvasive, *etc*) with each biomarker concentration at admission. A priori specified confounders; age (0–5 months, 6–11 months, ≥ 12 months), sex, MUAC, and enrollment site random effects were included in all multivariate models, and any of the additional variables associated with a biomarker (p < 0.05) in univariate analyses were also included in multivariable models.

### Mortality analysis

Cox proportional hazard models weighted for the CHAIN sampling strategy and the NCC design modelled mortality in the 30-days following hospital admission and the 180-days following discharge [3]. The 30-day period following admission was chosen because inpatient mortality is biased by discharge decisions. Crude 30-day models assessed associations between the ED biomarkers at admission and mortality. These models were then adjusted for admission variables a-priori identified confounder from the correlates analysis above (age, sex, MUAC, and enrollment site), in addition to other important risk factors for ED and mortality including LAZ, current breastfeeding (none, partial, exclusive), HIV status (infected/uninfected), invasive pathogen detection, caregiver reported recent diarrhea, and diagnoses of pneumonia, malaria and sepsis. Post-discharge models examined associations between discharge biomarkers concentrations and 180-day post-discharge mortality. Confounder adjusted post-discharge models included age, sex, discharge MUAC, enrollment site, discharge LAZ, current breastfeeding (none, partial, exclusive), HIV status (infected/uninfected), enteroinvasive pathogen detection at discharge, reported diarrhea at admission, and diagnoses of pneumonia, malaria and sepsis during admission. All analyses were performed in R (v3.6.1) with p < 0.05 (two-tailed) considered significant.

## Results

**Fig 1:**
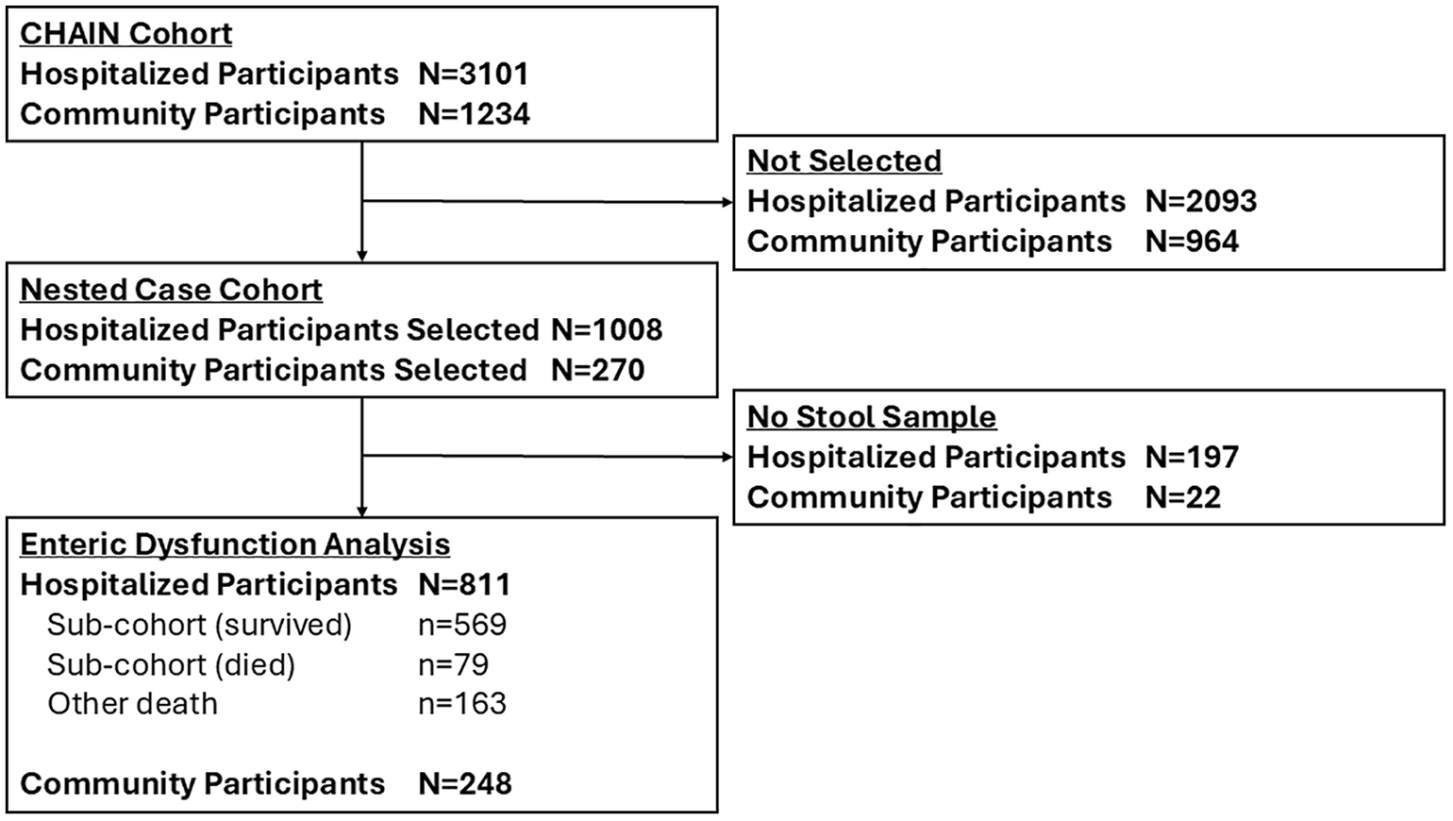
Study participant flow chart.

Among 811 included hospitalized children, 709 also had discharge data available (Fig 1). The median ages in months of hospitalized (10.6, IQR: 6.6-15.8) and community (12.2, IQR: 7.4-17.5) children were similar. Hospitalized children were less commonly exclusive breastfed (Table 1). Caregivers reported recent diarrhea among 457 (56%) children at admission. Compared to the community group, fewer hospitalized children were female and they had a higher prevalence of HIV infection, stunting, wasting and antibiotic exposure in the seven days prior to admission. By discharge, 92% of hospitalized children had received antibiotics. The median duration of hospitalization was 5 days (IQR 3–8).

**Table 1:** Baseline characteristics of study participants.

	Admission (N = 811)		Discharge (N = 709)		Community (N = 248)	
	n	%	n	%	n	%
**Site**						
Banfora	103	12.7	90	12.7	28	11.3
Blantyre	80	9.9	67	9.4	28	11.3
Kampala	109	13.4	94	13.3	24	9.7
Migori	93	11.5	65	9.2	25	10.1
Mbagathi	86	10.6	71	10	29	11.7
Kilifi	59	7.3	52	7.3	27	10.9
Karachi	95	11.7	95	13.4	27	10.9
Dhaka	108	13.3	100	14.1	30	12.1
Matlab	78	9.6	75	10.6	30	12.1
**Sex (Female)**	354	43.6	303	42.7	126	50.8
**Age**						
< 6 months	165	20.3	148	20.9	41	16.5
6-12 months	297	36.6	255	36	78	31.5
>12 months	349	43.0	306	43.2	129	52.0
**Any breastfeeding**	559	68.9	501	70.7	218	87.9
**Exclusive breastfeeding** ^**a**^	65	8.0	56	7.9	36	14.5
**Antibiotics in last 7 days**	365	45.0	653	92.1	34	13.7
**Stunted (LAZ < -2)**	439	54.1	367	51.8	83	33.5
**Severe wasting/kwashiorkor** ^**b**^	389	48.0	312	44.0	0	0.0
**Moderate wasting** ^**b**^	182	22.4	167	23.6	26	10.5
**Presenting Compliant**						
Diarrhea	457	56.4	385	54.3	–	–
Fever	594	73.2	530	74.8	–	–
Cough	401	49.4	403	56.8	–	–
≥1 IMCI Danger sign	353	43.5	280	39.5	–	–
**Diagnoses**						
Gastroenteritis	399	49.2	340	48.0	–	–
LRTI	321	39.6	92	13	–	–
Sepsis	109	13.4	279	39.4	–	–
Malaria (by RDT)	102	12.6	87	12.3	12	4.8
**HIV exposed uninfected**	58	7.2	47	6.6	19	7.7
**HIV infected**	54	6.7	33	4.7	3	1.2
**Household characteristics**						
Livestock ownership	275	33.9	244	34.4	109	44.0
Improve water source access	657	81.0	595	83.9	202	81.5
Improved sanitation access	614	75.7	559	78.8	178	71.8
Food insecure^c^	145	17.9	98	13.8	34	13.7

IMCI: Integrated Management of Childhood Illness, LAZ: Height-for-age Z-score, LRTI: lower respiratory tract infection; RDT: rapid diagnostic test; WHZ: Weight-for-height Z-score. ^a^Among infants under six months old, admission 59 (35.8%), discharge 53 (35.8%), community 30 (73.2%). ^b^Defined as per CHAIN study: Severe wasting/kwashiorkor (nutritional oedema, MUAC <11.5 cm if ≥6 months old, or MUAC <11 cm if <6 months old, Moderate wasting (MUAC <12.5 cm if ≥6 months old, or MUAC <12 cm if <6 months old) ^c^moderate to severe food insecurity.

### Pathogen prevalence

Detection of any enteropathogen was common, with prevalence of 84% at admission, 90% at discharge, and 61% among community children (Table 2). The prevalence of enteropathogens associated with dehydration at admission (29%) and discharge (27%) was comparable, but higher than among the community children (8%, p < 0.001 compared to admission). During hospitalization norovirus prevalence nearly doubled (p < 0.001), with no sites observing a decrease in norovirus detection, and seven sites having a higher prevalence at discharge than admission.

**Table 2:** Pathogen prevalence and ED biomarkers among included children, adjusted for sample selection into the nested case cohort.

	Admission			Discharge				Community			
	n	%^1^	(95% CI) ^1^	n	%^1^		(95% CI) ^1^	n	%^1^		(95% CI) ^1^
**Any pathogen**	**675**	**84.1**	**(82,87)**	**496**	**90.4**		**(88, 93)**	**151**	**60.9**		**(55, 67)**
**Dehydrating** ^**1**^	**209**	**28.9**	**(26, 32)**	**178**	**27.0**		**(24, 30)**	**20**	**8.1**		**(5, 12)**
Adenovirus	44	5.7	(4, 7)	85	3.0		(2, 4)	7	2.8		(1, 6)
Astrovirus	24	3.0	(2, 4)	47	7.0		(5, 9)	2	0.8		(0, 3)
Norovirus	53	7.1	(5, 9)	85	12.4		(10, 15)	10	4.0		(2, 7)
Rotavirus	111	15.8	(13, 18)	54	7.9		(6, 10)	1	0.4		(0, 2)
**Invasive** ^**2**^	**576**	**69.4**	**(66, 73)**	**391**	**59.6**		**(56, 63)**	**151**	**60.9**		**(55, 67)**
*Aeromonas*	4	0.1	(0, 1)	2	0.0		(0, 0)	3	1.2		(0, 3)
*Campylobacter*	211	25.7	(23, 29)	86	13		(11, 16)	49	19.8		(15, 25)
EAEC	444	52.9	(49, 56)	331	50.1		(47, 54)	99	39.9		(34, 46)
Atypical EPEC	80	9.8	(8, 12)	37	4.9		(3, 6)	22	8.9		(6, 13)
Typical EPEC	113	11.9	(10, 14)	50	6.8		(5, 9)	19	7.7		(5, 12)
*Salmonella*	12	1.6	(0, 2)	9	1.2		(0, 2)	1	0.4		(0, 2)
*Shigella*	100	11.1	(9, 13)	26	3.6		(2, 5)	18	7.3		(5, 11)
**ETEC**	142	17.8	(15, 20)	58	8.9		(7, 10)	27	10.9		(8, 15)
***Giardia***	56	8.0	(6, 10)	46	7.9		(6, 10)	26	10.5		(7, 15)
***Cryptosporidium***	94	10.1	(8, 12)	59	7.9		(6, 11)	13	5.2		(3, 9)
	**n**	**median**	**(IQR)**	**n**	**median**		**(IQR)**	**n**	**median**		**(IQR)**
**Myeloperoxidase (ng/ml)**	681	2290	(868, 6052)	558		1380	(611, 3193)	248		2268	(1051, 5421)
**Calprotectin (ug/ml)**	652	215	(77, 746)	549		170	(68, 369)	242		252	(124, 691)
**α-1-antitrypsin (mg/l)**	678	121	(49, 303)	555		144	(69, 275)	247		201	(100, 412)

^1^Percentages and 95% Confidence intervals have been weighted for the nested case cohort sample selection. ^2^Based on Kosek et al (2017), EAEC: enteroaggregative *E. coli*, EPEC: enteropathogenic *E. coli*, ETEC: enterotoxigenic *E. coli*, IQR: inter-quartile range

Enteroinvasive pathogens were most prevalent at admission (69%) compared to discharge from hospital (60%, p < 0.001) or the community (61%, p = 0.004). Several invasive pathogens had non-significant declines during hospitalization to levels comparable or slightly below the community including *Campylobacter* (26% admission, 13% discharge, 20% community), atypical EPEC (10% admission, 5% discharge, 9% community), and *Shigella* (11% admission, 4% discharge, 7% community).

ETEC was more prevalent at admission (18%) than discharge (9%, p < 0.001) and community groups (11%, p = 0.014). *Cryptosporidium* was more prevalent at admission (10%, p = 0.033), but similar at discharge (8%, p = 0.100), compared to community children (5%). Finally, *Giardia* was similar at admission (8%, p = 0.060) and discharge (8%, p = 0.080) compared to the community group (11%).

### ED biomarkers

**Fig 2:**
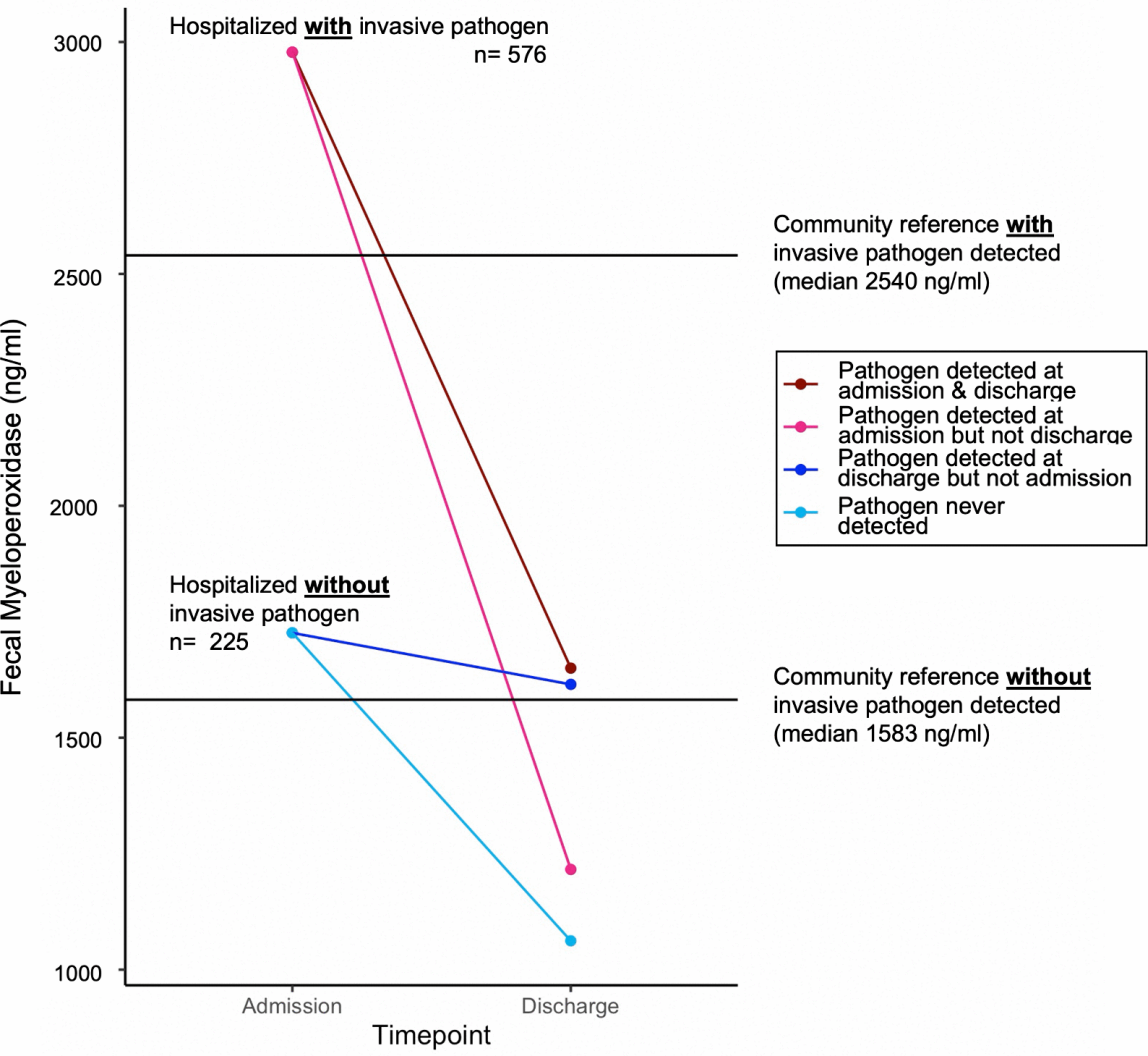
Median myeloperoxidase concentrations at admission and discharge from hospital compared to community levels, stratified by the detection of invasive pathogens.

Median admission fecal myeloperoxidase concentrations among hospitalized children were higher than in the community (p = 0.014), but fell below community levels at discharge (p < 0.001, Fig 2). Admission calprotectin among hospitalized children was comparable with the community (p = 0.351), but fell below community levels by discharge (p = 0.007, S1 Appendix). α-1-antitrypsin concentrations among hospitalized children were lower at admission (p = 0.049), but similar at discharge (p = 0.380), as compared to the community.

### Sensitivity analyses

Children with reported diarrhea had higher rotavirus, norovirus, typical EPEC and *Shigella* prevalence at admission, but fewer detections of *Giardia* than children without diarrhea. However, the trend of decreased pathogen prevalence between admission and discharge, particularly invasive enteropathogens, was similar among hospitalized children with and without reported diarrhea (S1 Appendix).

The decline in myeloperoxidase and calprotectin across the admission was consistent in hospitalized children with and without reported diarrhea. However, higher median α-1-antitrypsin concentrations during admission were isolated to children with reported diarrhea at admission. Sensitivity analyses including only children with both admission and discharge samples showed very similar trends to the main analysis (S1 Appendix).

### Correlates of ED biomarkers at admission

In crude models, myeloperoxidase concentrations were positively associated with younger age, oedema, lower MUAC, lower LAZ, absence of dehydrating pathogens, enteroinvasive pathogen detection, and being hospitalized (Table 3). Multivariable models found MUAC, LAZ, age, and invasive enteropathogen detection remained associated with myeloperoxidase. Invasive pathogen detection was associated with a 0.28 SD (95% CI: 0.10, 0.46; p = 0.003) higher fecal myeloperoxidase. A one cm smaller MUAC was associated with 0.07 SD (95% CI: 0.12, 0.03; p = 0.002) higher myeloperoxidase, while a one SD decrease in LAZ had a corresponding 0.06 SD (95% CI: 0.10, 0.01; p = 0.014) higher myeloperoxidase. Children who were 6–11 months old had a 0.20 SD (95% CI: 0.38, 0.02, p = 0.026) lower myeloperoxidase than those ≥12 months old. Correlates of biomarkers among the community group are provided for comparison (S1 Appendix).

**Table 3:** Correlates of ED fecal biomarkers.

	Myeloperoxidase				Calprotectin				α-1-antitrypsin			
	Unadjusted		Adjusted^1^		Unadjusted		Adjusted^1^		Unadjusted		Adjusted^1^	
	Coef	(95% CI)	Coef	(95% CI)	Coef	(95% CI)	Coef	(95% CI)	Coef	(95% CI)	Coef	(95% CI)
**Hospital vs community**	**0.16**	**(0.02, 0.31)**	0.09	(-0.10, 0.28)	0.08	(-0.07, 0.22)			-0.13	(-0.28, 0.01)		
**Hospitalized cohort only**												
Oedema	**0.24**	**(0.01, 0.48)**	0.14	(-0.10, 0.38)	0.02	(-0.24, 0.21)	--		-0.19	(-0.42, 0.03)	--	
MUAC	**-0.07**	**(-0.12, -0.02)**	**-0.07**	**(-0.12, -0.03)**	**-0.06**	**(-0.10, -0.01)**	**-0.06**	**(-0.10, -0.01)**	0.04	(-0.00, 0.08)	--	
Height-for-age	**-0.07**	**(-0.11, -0.02)**	**-0.06**	**(-0.10, -0.01)**	**-0.06**	**(-0.10, -0.01)**	**-0.05**	**(-0.09, -0.01)**	0.03	(-0.01, 0.07)	--	
Breastfeeding												
Partial vs exclusive	0.04	(-0.32, 0.25)	--		-0.13	(-0.40, 0.13)	--		0.13	(-0.13, 0.40)	--	
None vs exclusive	-0.01	(-0.20, 0.18)	--		-0.09	(-0.27, 0.09)	--		**-0.22**	**(-0.40, -0.04)**	**-0.13**	**(-0.15, -0.40)**
Months of age												
<6 vs>12	-0.11	(-0.32, 0.11)	--		-0.09	(-0.30, 0.12)	--		-0.11	(-0.31, 0.10)	--	
6-12 vs > 12	**-0.23**	**(-0.42, -0.01)**	**0.20 (-0.38, -0.02)**		-0.11	(-0.28, 0.07)	--		**-0.29**	**(-0.46, -0.11)**	--	
Reported diarrhea	-0.09	(-0.27, 0.08)	--		0.03	(-0.14, 0.20)	--		**-0.57**	**(-0.73, -0.41)**	**-0.43**	**(-0.59, -0.27)**
Diagnosis sepsis	0.16	(-0.10, 0.41)	--		0.21	(-0.04, 0.45)	--		-0.10	(-0.35, 0.14)	--	
Diagnosis pneumonia	0.04	(-0.13, 0.41)	--		0.09	(-0.08, 0.26)	--		**0.31**	**(0.15, 0.49)**	0.16	(-0.08, 0.41)
Diagnosis malaria^2^	-0.24	(-0.52, 0.04)	--		-0.04	(-0.31, 0.22)	--		**0.23**	**(0.01, 0.48)**	0.16	(-0.00, 0.31)
Dehydrating pathogen	**-0.25**	**(-0.44, -0.06)**	-0.16	(-0.35, 0.03)	-0.18	(-0.37, 0.00)	--		**-0.28**	**(-0.46, -0.09)**	-0.15	(-0.32, 0.03)
Invasive pathogen	**0.32**	**(0.14, 0.49)**	**0.28**	**(0.10, 0.46)**	**0.32**	**(0.15, 0.50)**	**0.29**	**(0.11, 0.47)**	0.04	(-0.13, 0.22)	--	

^1^Models were adjusted for invasive pathogens, dehydrating pathogens, reported diarrhea, age group, sex and mid-upper arm circumference with site as a random effect. Height-for-age z-score models did not include MUAC due to co-linearity. ^2^Based on positive malaria rapid diagnostic. MUAC: mid upper arm circumference.

Similarly, lower MUAC, lower LAZ and invasive pathogen detection were associated with calprotectin in crude models and remained associated in multivariable models: invasive pathogen detection (0.29 SD; 95% CI: 0.11, 0.47; p = 0.002), lower MUAC (0.06 SD, 95% CI: 0.10, 0.01, p = 0.012), and lower LAZ (0.05 SD, 95% CI: 0.09, 0.01, p = 0.023). Among the invasive enteropathogens, both *Shigella* spp*.* and EAEC were associated with increased myeloperoxidase and calprotectin (S1 Appendix).

Higher α-1-antitrypsin levels were associated with younger age, not breastfeeding, and having a pneumonia or malaria diagnosis, while reported diarrhea or detection of dehydrating pathogens were associated with lower α-1-antitrypsin. In multivariable regression, only reported diarrhea remained significantly associated α-1-antitrypsin (-0.43 SD; 95%CI: -0.59, -0.27; p < 0.001).

### Mortality

Crude models found admission myeloperoxidase and calprotectin concentrations were associated with mortality in the 30-days following hospital admission (Table 4). However, after adjustment for confounding, neither biomarker remained associated with 30-day mortality. α-1-antitrypsin at admission was not associated with subsequent 30-day mortality in either crude or adjusted models

**Table 4:** Association between enteric dysfunction biomarkers and mortality in the hospital cohort.

		Crude				Adjusted^1^			
		HR	(95% CI)		P-value	HR	(95% CI)		p-value
**Admission** 30-day mortality^2^	MPO	**1.34**	**(1.12,**	**1.59)**	**0.001**	1.11	0.90	1.38	0.324
	CAL	**1.20**	**(1.02,**	**1.42)**	**0.033**	0.99	0.80	1.23	0.957
	AAT	1.09	(0.92,	1.29)	0.310	1.07	0.86	1.35	0.529
**Discharge** 180-day mortality^3^	MPO	**1.31**	**(1.09,**	**1.56)**	**0.004**	1.16	0.94	1.42	0.161
	CAL	**1.19**	**(0.99,**	**1.42)**	**0.069**	**1.36**	**1.07**	**1.73**	**0.011**
	AAT	0.99	(0.80,	1.22)	0.931	0.97	0.76	1.23	0.792

^1^Models were adjusted for invasive pathogens, recent diarrhea, age group, sex, breastfeeding, mid-upper arm circumference, height-for-age z-score, site and diagnoses of pneumonia, diarrhea and malaria. ^2^Mortality model for admission to 30 days. ^3^Discharge to 180-days post-discharge.

Abbreviations: MPO: Myeloperoxidase, CAL: calprotectin, AAT: α-1-antitrypsin, HR: Hazard ratio, CI: Confidence Interval. EAEC: entero-aggregative *E. coli*, EPEC: enteropathogenic *E. coli*, ETEC: enter-toxigenic *E. coli*,

Myeloperoxidase and calprotectin at discharge were associated with 180-day mortality following discharge in crude models. Discharge myeloperoxidase was not associated with mortality after adjustment, but a one SD increase in calprotectin at discharge was associated with 36% (hazard ratio 1.36, 95% CI: 1.07,1.72, p = 0.011) mortality increase. α-1-antitrypsin at discharge was not associated with 180-day post-discharge mortality in either crude or adjusted models.

## Discussion

Biomarkers of enteric inflammation, including fecal myeloperoxidase and calprotectin, decreased during hospitalization to levels significantly lower than community peers, although the majority of children at admission, discharge (and in the community) had levels above what is considered normal for age (2000ng/ml for myeloperoxidase and 77 ug/ml for calprotectin) [22,23]. We also observed a decrease in invasive enteropathogen prevalence during hospitalization and the detection of invasive enteropathogens was strongly associated with enteric inflammation biomarkers among hospitalized children. Finally, fecal calprotectin at discharge from hospital was associated with post-discharge mortality in the adjusted model. Collectively, these data support the hypothesis that a high prevalence of invasive enteropathogens promotes enteric inflammation among acutely unwell children.

There was a high prevalence of enteropathogens among the community children, but an even greater burden among children admitted to hospital. The individual pathogen prevalence in our analysis aligned well with the consensus understanding of diarrhea etiology and asymptomatic carriage in LMICs [9,16,24–26]. EAEC and *Campylobacter* were commonly detected but were not specifically associated with diarrhea, while rotavirus and *Shigella* were most prevalent at admission among children presenting with diarrhea. *Giardia* appeared to be more common among community than hospitalized children, as has been observed in other settings, although the difference was not significant [27]. The prevalence of many pathogens substantially decreased between admission and discharge and this decline was prominent among *Campylobacter, Shigella*, and ETEC. Children enrolled in CHAIN were hospitalized for an average of five days, and 92% of admitted children received antibiotics. Penicillin, cephalosporin and gentamicin use was common and is likely to have played a role in the decline of bacterial enteropathogens during hospitalization. Changes in water and sanitation exposures and food types and preparation practices may also have contributed to declines in enteropathogens during hospitalization, although the evidence for the effectiveness of these interventions in community setting is mixed [28]. The lower fecal myeloperoxidase and calprotectin concentrations at discharge compared to the community may be partially attributable to the decline in invasive enteropathogens, or the direct effects of antibiotic, nutritional and environmental exposures common to inpatient management.

Enteric inflammatory biomarkers have been associated with increased morbidity, systemic inflammation, reduced vaccine responsiveness, and linear or ponderal growth delays [4,5,29]. Both systemic inflammation and poor nutritional status have been shown to be strongly predictors of post-discharge mortality [3,30–32]. It is possible that post-discharge morbidity and mortality could be reduced by therapeutics that help children avoid re-colonization with enteroinvasive pathogens or extend the period of reduced enteric inflammation after hospitalization by promoting nutrient uptake and decreasing systemic inflammation. A pilot trial in Kenya demonstrated that intestinal immunosuppression with the aminosalicylate mesalazine is safe among children with severe acute malnutrition and may be beneficial in reducing enteric inflammation [33]. A recent trial of enteric interventions at hospital discharge among children with severe acute malnutrition found that teduglutide, a glucagon-like peptide that promotes mucosal regeneration, reduced enteric inflammation, while oral budesonide, a corticosteroid active in the gut, was associated with reduced plasma C-reactive protein [34].

The observed increase in levels of fecal α-1-antitrypsin, an enteric permeability biomarker, during hospitalization was in the opposite direction compared to the enteric inflammatory biomarkers. However, this trend was isolated to children who had presented with diarrhea. A dilutional effect of diarrhea on α-1-antitrypsin concentrations at admission is highly likely, supported by our finding of a strong association between lower α-1-antitrypsin concentrations and dehydrating enteropathogens. However, α-1-antitrypsin did appear to be raised among children with either pneumonia or malaria. *P. Falciparum* malaria sequesters in intestinal capillaries and is thought to cause local hypoxia and increased permeability which may explain the association with α-1-antitrypsin [35,36].

Norovirus prevalence increased during hospitalization across multiple sites. Norovirus acquisition was most common among children admitted with diarrhea, which may indicate missed norovirus detections at admission or that norovirus is adept at infecting children with recent diarrhea. Children with diarrhea are often accommodated in distinct units within pediatric wards which may facilitate nosocomial norovirus acquisition. This finding suggests that norovirus testing for norovirus and isolation of infected patients could prevent nosocomial spread on paediatric units in LMICs.

The associations between ED biomarkers at admission and mortality were strongly confounded by factors such as MUAC, age and background illnesses suggesting ED at admission is either not a driver of mortality, that its contribution is masked by other factors of more substantial magnitude, or that it acts as a mediator for known risks factors of mortality. A case-control analysis among unwell severely malnourished children in Malawi and Kenya also found no association between fecal myeloperoxidase or calprotectin and inpatient deaths, [37] perhaps suggesting ED is not a strong determinant of inpatient mortality.

We did find that higher discharge calprotectin levels were associated with post-discharge mortality, potentially indicating that enteric inflammation undermines recovery in the post-discharge period. It is interesting to note that while the mean calprotectin was lower among children at admission, and especially at discharge, compared to community peers, the mean admission and discharge calprotectin concentrations were above levels considered within normal limits for age and even above levels considered sensitive and specific for inflammatory bowel disease [23, 38]. The association calprotectin-post-discharge mortality association suggests that enteric inflammation interplays with other factors among vulnerable children to increase the risk of death.

This analysis included data from nine hospitals in six countries and leveraged a broad panel of enteropathogens and ED biomarkers. However, the analysis has several limitations. These data were observational limiting our ability to infer causality. For example, systemic inflammation is known to be associated with post-discharge outcomes [31,32,39]. While systemic inflammation is considered to be a downstream consequence of enteric inflammation, [12,40] it is plausible that systemic inflammation may contribute to enteric inflammation. We did adjust for major diagnoses associated with systemic inflammation – namely sepsis, pneumonia, and malaria. However, systemic inflammation, especially due to other etiologies could be a residual confounder in the association between discharge calprotectin and mortality. Our cohort oversampled children with wasting to gain insights into patients with a high risk of mortality, but this may limit the generalizability or our findings to lower risk populations. Stool for measuring the concentration of ED biomarkers was not available for all children, which may have introduced selection bias. Diarrhea may have caused some dilution effect in ED biomarkers, although the trends observed in our analysis were also present among children without diarrhea. Finally, molecular enteropathogen diagnostics are highly sensitive, and detection of bacterial DNA after a course of antibiotics may be due to remnant nucleic acid rather than live bacteria. This would suggest our results could overestimate enteropathogen prevalence at discharge. Ultimately, interventional trials of therapeutics are needed to determine the clinical significance of ED in the post-discharge period.

## Conclusions

Enteropathogens were common among community children, but even more prevalent among those admitted to hospital. During the hospitalization, the prevalence of bacterial pathogens and the concentration of enteric inflammatory biomarkers decreased to the point that children at discharge had lower enteric inflammation and fewer bacterial pathogen detections than comparable children in the community. However, most children had fecal calprotectin concentrations at discharge above levels considered within normal for age, and it was associated with post-discharge mortality. Collectively, these results suggest that at the point of discharge children may have relatively lower enteropathogen burden and less severe enteric inflammation and it is possible extending this period of relative enteric health may be opportunity to improve post-discharge outcomes.

## Supporting information

S1 Appendix Additional methods and results as cited in the main text. (DOCX)

S1 Checklist Inclusivity in global research checklist. (DOCX)

## References

[1] Wiens MO, Pawluk S, Kissoon N, Kumbakumba E, Ansermino JM, Singer J, et al. Pediatric post-discharge mortality in resource poor countries: A systematic review. PLoS One. 2013;8(6):e66698. doi: 10.1371/journal.pone.0066698 23825556 PMC3692523

[2] Nemetchek BR, Liang LD, Kissoon N, Ansermino JM, Kabakyenga J, Lavoie PM, et al. Predictor variables for post-discharge mortality modelling in infants: A protocol development project. Afr Health Sci. 2018;18(4):1214–25. doi: 10.4314/ahs.v18i4.43 30766588 PMC6354852

[3] Childhood Acute Illness and Nutrition (CHAIN) Network. Childhood mortality during and after acute illness in Africa and south Asia: A prospective cohort study. Lancet Glob Health. 2022;10(5):e673–84. doi: 10.1016/S2214-109X(22)00118-8 35427524 PMC9023747

[4] Keusch GT, Denno DM, Black RE, Duggan C, Guerrant RL, Lavery JV, et al. Environmental enteric dysfunction: Pathogenesis, diagnosis, and clinical consequences. Clinical infectious diseases: An official publication of the Infectious Diseases Society of America. 2014;59 Suppl 4: S207–12. doi: 10.1093/cid/ciu485 PMC4481570 25305288

[5] Tickell KD, Atlas HE, Walson JL. Environmental enteric dysfunction: A review of potential mechanisms, consequences and management strategies. BMC Med. 2019;17(1):181. doi: 10.1186/s12916-019-1417-3 31760941 PMC6876067

[6] Prendergast AJ, Humphrey JH. The stunting syndrome in developing countries. Paediatr Int Child Health. 2014;34(4):250–65. doi: 10.1179/2046905514Y.0000000158 25310000 PMC4232245

[7] Kosek MN, MAL-ED Network Investigators. Causal pathways from enteropathogens to environmental enteropathy: Findings from the MAL-ED birth cohort study. EBioMedicine. 2017;18:109–17. doi: 10.1016/j.ebiom.2017.02.024 28396264 PMC5405169

[8] Iqbal NT, Syed S, Kabir F, Jamil Z, Akhund T, Qureshi S, et al. Pathobiome driven gut inflammation in Pakistani children with Environmental Enteric Dysfunction. PLoS One. 2019;14(8):e0221095. doi: 10.1371/journal.pone.0221095 31442248 PMC6707605

[9] Iqbal NT, Lawrence S, Ahmed T, Chandwe K, Fahim SM, Houpt ER, et al. Enteric pathogens relationship with small bowel histologic features of environmental enteric dysfunction in a multicountry cohort study. Am J Clin Nutr. 2024;120 Suppl 1(Suppl 1):S84–93. doi: 10.1016/j.ajcnut.2024.02.026 39300666 PMC13168960

[10] Jamil Z, Iqbal NT, Idress R, Ahmed Z, Sadiq K, Mallawaarachchi I, et al. Gut integrity and duodenal enteropathogen burden in undernourished children with environmental enteric dysfunction. PLoS Negl Trop Dis. 2021;15(7):e0009584. doi: 10.1371/journal.pntd.0009584 34264936 PMC8352064

[11] Kabir F, Iqbal J, Jamil Z, Iqbal NT, Mallawaarachchi I, Aziz F, et al. Impact of enteropathogens on faltering growth in a resource-limited setting. Front Nutr. 2023;9:1081833. doi: 10.3389/fnut.2022.1081833 36704796 PMC9871909

[12] Harper KM, Mutasa M, Prendergast AJ, Humphrey J, Manges AR. Environmental enteric dysfunction pathways and child stunting: A systematic review. PLoS Negl Trop Dis. 2018;12(1):e0006205. doi: 10.1371/journal.pntd.0006205 29351288 PMC5792022

[13] Prendergast AJ, Humphrey JH, Mutasa K, Majo FD, Rukobo S, Govha M, et al. Assessment of environmental enteric dysfunction in the SHINE Trial: Methods and challenges. Clinical Infectious Diseases. 2015;61:S726-32. doi: D 10.1093/cid/civ848 PMC4657593 26602300

[14] Sturgeon JP, Tome J, Dumbura C, Majo FD, Ngosa D, Mutasa K, et al. Inflammation and epithelial repair predict mortality, hospital readmission, and growth recovery in complicated severe acute malnutrition. Sci Transl Med. 2024;16(736):eadh0673. doi: 10.1126/scitranslmed.adh0673 38416844 PMC7615785

[15] Allen CAD, Ghate A, Njunge JM, Gartner L, Diallo AH, Lancioni C. Plasma lipopolysaccharide levels predict mortality in acutely ill children in low- and middle-income countries. Nature Communications. 2025;16:10787. doi: 10.1038/s41467-025-65429-0 PMC12663156 41315218

[16] Kosek M, Haque R, Lima A, Babji S, Shrestha S, Qureshi S, et al. Fecal markers of intestinal inflammation and permeability associated with the subsequent acquisition of linear growth deficits in infants. Am J Trop Med Hyg. 2013;88(2):390–6. doi: 10.4269/ajtmh.2012.12-0549 23185075 PMC3583335

[17] Njunge JM, Tickell K, Diallo AH, Sayeem Bin Shahid ASM, Gazi MA, Saleem A, et al. The Childhood Acute Illness and Nutrition (CHAIN) network nested case-cohort study protocol: A multi-omics approach to understanding mortality among children in sub-Saharan Africa and South Asia. Gates Open Res. 2022;6:77. doi: 10.12688/gatesopenres.13635.2 36415883 PMC9646488

[18] Immundiagnostik A. IDK MPO ELSIA. https://fnkprddata.blob.core.windows.net/domestic/data/datasheet/IMD/KR6630.pdf. 2019.

[19] Immundiagnostik AG. IDK a1-Antitrypsin ELISA. https://www.immundiagnostik.com/media/pages/portfolio/testkits/k-6750/a7689eb802-1774004732/k6750_2025-07-25_a1-antitrypsin_stuhl.pdf. 2025.

[20] Immundiagnostik AG. IDK Calprotectin ELISA. https://www.immundiagnostik.com/media/pages/portfolio/testkits/kr6927/0e31900440-1774004715/kr6927_2024-08-01_idk_calprotectin_stuhl_1h.pdf. 2024.

[21] Liu J, Kabir F, Manneh J, Lertsethtakarn P, Begum S, Gratz J, et al. Development and assessment of molecular diagnostic tests for 15 enteropathogens causing childhood diarrhoea: A multicentre study. Lancet Infect Dis. 2014;14(8):716–24. doi: 10.1016/S1473-3099(14)70808-4 25022434

[22] Otiti MI, Dodd J, K’Oloo A, June M, Chomba M, Wang D, et al. Environmental enteric dysfunction, systemic inflammation, growth hormones, and linear growth in infants in western Kenya: A prospective observational cohort study. Am J Clin Nutr. 2026;123(1):101095. doi: 10.1016/j.ajcnut.2025.10.012 41235975

[23] Davidson F, Lock RJ. Paediatric reference ranges for faecal calprotectin: A UK study. Ann Clin Biochem. 2017;54(2):214–8. doi: 10.1177/0004563216639335 27141011

[24] Kotloff KL, Blackwelder WC, Nasrin D, Nataro JP, Farag TH, van Eijk A, et al. The Global Enteric Multicenter Study (GEMS) of diarrheal disease in infants and young children in developing countries: Epidemiologic and clinical methods of the case/control study. Clin Infect Dis. 2012;55 Suppl 4(Suppl 4):S232-45. doi: 10.1093/cid/cis753 23169936 PMC3502307

[25] Platts-Mills JA, Babji S, Bodhidatta L, Gratz J, Haque R, Havt A. Pathogen-specific burdens of community diarrhoea in developing countries: A multisite birth cohort study (MAL-ED). MAL-ED. 2015. doi: 10.1001/jama.2016.12345 PMC7328884 26202075

[26] Praharaj I, Revathy R, Bandyopadhyay R, Benny B, Azharuddin Ko M, Liu J, et al. Enteropathogens and gut inflammation in asymptomatic infants and children in different environments in Southern India. Am J Trop Med Hyg. 2018;98(2):576–80. doi: 10.4269/ajtmh.17-0324 29231154 PMC5929183

[27] Muhsen K, Levine MM. A systematic review and meta-analysis of the association between Giardia lamblia and endemic pediatric diarrhea in developing countries. Clinical Infectious Diseases: An Official Publication of the Infectious Diseases Society of America. 2012;55(Suppl 4):S271-93. doi: 10.1093/cid/cis762 PMC3502312 23169940

[28] Gough EK, Moulton LH, Mutasa K, Ntozini R, Stoltzfus RJ, Majo FD, et al. Effects of improved water, sanitation, and hygiene and improved complementary feeding on environmental enteric dysfunction in children in rural Zimbabwe: A cluster-randomized controlled trial. PLoS Negl Trop Dis. 2020;14: e0007963. doi: 10.1371/journal.pntd.0007963 PMC7046282 32059011

[29] Arndt MB, Cantera JL, Mercer LD, Kalnoky M, White HN, Bizilj G, et al. Validation of the Micronutrient and Environmental Enteric Dysfunction Assessment Tool and evaluation of biomarker risk factors for growth faltering and vaccine failure in young Malian children. PLoS Negl Trop Dis. 2020;14(9):e0008711. doi: 10.1371/journal.pntd.0008711 32997666 PMC7549819

[30] Childhood Acute Illness and Nutrition (CHAIN) Network. Characterising paediatric mortality during and after acute illness in Sub-Saharan Africa and South Asia: A secondary analysis of the CHAIN cohort using a machine learning approach. EClinicalMedicine. 2023;57:101838. doi: 10.1016/j.eclinm.2023.101838 36825237 PMC9941052

[31] Njunge JM, Gwela A, Kibinge NK, Ngari M, Nyamako L, Nyatichi E, et al. Biomarkers of post-discharge mortality among children with complicated severe acute malnutrition. Sci Rep. 2019;9(1):5981. doi: 10.1038/s41598-019-42436-y 30979939 PMC6461700

[32] Gonzales GB, Njunge JM, Gichuki BM, Wen B, Potani I, Voskuijl W, et al. Plasma proteomics reveals markers of metabolic stress in HIV infected children with severe acute malnutrition. Sci Rep. 2020;10(1):11235. doi: 10.1038/s41598-020-68143-7 32641735 PMC7343797

[33] Jones KD, Hunten-Kirsch B, Laving AM, Munyi CW, Ngari M, Mikusa J. Mesalazine in the initial management of severely acutely malnourished children with environmental enteric dysfunction: A pilot randomized controlled trial. BMC Med. 2014;12(1):133. doi: 10.1186/s12916-014-0133-2 25189855 PMC4243388

[34] Chandwe K, Bwakura-Dangarembizi M, Amadi B, Tawodzera G, Ngosa D, Dzikiti A, et al. Malnutrition enteropathy in Zambian and Zimbabwean children with severe acute malnutrition: A multi-arm randomized phase II trial. Nat Commun. 2024;15(1):2910. doi: 10.1038/s41467-024-45528-0 38632262 PMC11024201

[35] Zeng H, He X, Tuo Q-H, Liao D-F, Zhang G-Q, Chen J-X. LPS causes pericyte loss and microvascular dysfunction via disruption of Sirt3/angiopoietins/Tie-2 and HIF-2α/Notch3 pathways. Sci Rep. 2016;6:20931. doi: 10.1038/srep20931 26868537 PMC4751495

[36] Brenchley JM, Douek DC. Microbial translocation across the GI tract. Annu Rev Immunol. 2012;30:149–73. doi: 10.1146/annurev-immunol-020711-075001 22224779 PMC3513328

[37] Wen B, Farooqui A, Bourdon C, Tarafdar N, Ngari M, Chimwezi E, et al. Intestinal disturbances associated with mortality of children with complicated severe malnutrition. Commun Med (Lond). 2023;3(1):128. doi: 10.1038/s43856-023-00355-0 37773543 PMC10541881

[38] Diamanti A, Panetta F, Basso MS, Forgione A, Colistro F, Bracci F, et al. Diagnostic work-up of inflammatory bowel disease in children: the role of calprotectin assay. Inflamm Bowel Dis. 2010;16(11):1926–30. doi: 10.1002/ibd.21257 20310017

[39] Njunge JM, Gonzales GB, Ngari MM, Thitiri J, Bandsma RHJ, Berkley JA. Systemic inflammation is negatively associated with early post discharge growth following acute illness among severely malnourished children - A pilot study. Wellcome Open Res. 2021;5:248. doi: 10.12688/wellcomeopenres.16330.2 33969227 PMC8080977

[40] Caballero-Mateos AM, Brunet-Mas E, Gros B. Systemic consequences of inflammatory bowel disease beyond immune-mediated manifestations. J Clin Med. 2025;14(22):7984. doi: 10.3390/jcm14227984 41303019 PMC12653986

