# Supplementary material for: Enteric dysfunction and enteropathogens among hospitalized south Asian and sub-Saharan African children
Source: PLOS Glob Public Health. 2026 Jul 24;6(7):e0006873. doi: 10.1371/journal.pgph.0006873 (PMC13399345; doi:10.1371/journal.pgph.0006873)
Supplement: S1 Appendix — (DOCX) [file pgph.0006873.s001.docx]

**S1 Appendix**

**Table A. TAC card target**

|  | **Locus or Protein encoded** |
| --- | --- |
| *Aeromonas* | Aerolysin |
| *Campylobacter*_pan | *cpn60* |
| EAEC (Enteroaggregative *E. coli*)_aaiC | *aaiC* |
| EAEC (Enteroaggregative *E. coli*)_aatA | *aatA* |
| EPEC, atypical (Enteropathogenic *E. coli*)_bfpA | *bfpA* |
| EPEC. typical (Enteropathogenic *E. coli*)_eae | *eae* |
| ETEC (Enterotoxigenic *E. coli*)_LT | LT |
| ETEC (Enterotoxigenic *E. coli*)_STh | STh |
| ETEC (Enterotoxigenic *E. coli*)_STp | STp |
| *Salmonella enterica* | *ttr* |
| *Shigella*_EIEC (Enteroinvasive *E. coli*) | *ipaH* |
| STEC (Shiga toxin-producing *E. coli*)_stx1 | *stx1* |
| STEC (Shiga toxin-producing *E. coli*)_stx2 | *stx2* |
| *Vibrio cholerae* | *hlyA* |
| Adenovirus 40/41 | Fiber gene |
| Astrovirus | Capsid |
| Norovirus GI | *ORF1-2* |
| Norovirus GII | *ORF1-2* |
| Rotavirus | *NSP3* |
| *Cryptosporidium* | 18S rRNA |
| *Giardia lamblia* | 18S rRNA |
|  |  |
|  |  |
|  |  |
|  |  |

**Figure A. Median calprotectin concentrations at admission and discharge from hospital compared to community levels, stratified by the detection of invasive pathogens**

**
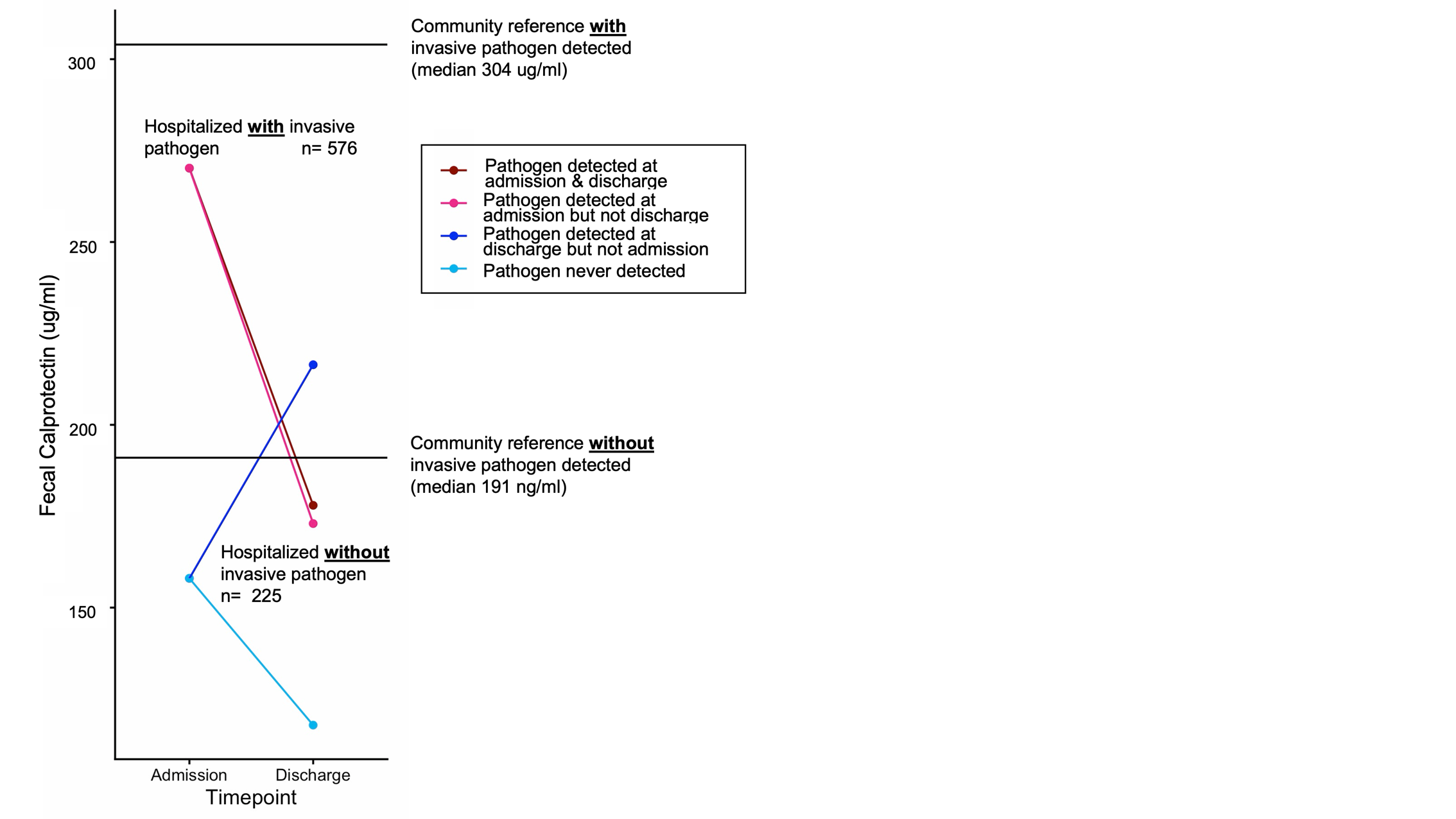
**

**Table B. Pathogen prevalence among children with diarrhea at admission compared to the community children**

|  | **Admission** | | | **Discharge** | | | **Community** | | |
| --- | --- | --- | --- | --- | --- | --- | --- | --- | --- |
|  | **n** | **%** | **(95% CI)** | **n** | **%** | **(95% CI)** | **N** | **%** | **(95% CI)** |
| **Dehydrating^1^** | **167** | **36.5** | **(32, 41)** | **131** | **36** | **(31, 41)** | **167** | **36.5** | **(32, 41)** |
| Adenovirus | 34 | 7.4 | (5, 10) | 13 | 3.6 | (2, 6) | 34 | 7.4 | (5, 10) |
| Astrovirus | 16 | 3.5 | (2, 6) | 40 | 11 | (8, 15) | 2 | 0.8 | (0, 3) |
| Norovirus | 34 | 7.4 | (5, 10) | 61 | 16.8 | (13, 21) | 10 | 4.0 | (2, 7) |
| Rotavirus | 106 | 23.2 | (20, 27) | 44 | 12.1 | (9, 16) | 1 | 0.4 | (0, 2) |
| **Invasive^1^** | **329** | **72** | **(68, 76)** | **246** | **67.6** | **(63, 72)** | **151** | **60.9** | **(55, 67)** |
| *Aeromonas* | 2 | 0.4 | (0, 2) | 2 | 0.5 | (0, 2) | 3 | 1.2 | (0, 3) |
| *Campylobacter* | 121 | 26.5 | (23, 31) | 49 | 13.5 | (10, 17) | 49 | 19.8 | (15, 25) |
| EAEC | 255 | 55.8 | (51, 60) | 213 | 58.5 | (53, 63) | 99 | 39.9 | (34, 46) |
| Atypical EPEC | 39 | 8.5 | (6, 11) | 19 | 5.2 | (3, 8) | 22 | 8.9 | (6, 13) |
| Typical EPEC | 80 | 17.5 | (14, 21) | 40 | 11 | (8, 15) | 19 | 7.7 | (5, 12) |
| *Salmonella* | 5 | 1.1 | (0, 3) | 5 | 1.4 | (1, 3) | 1 | 0.4 | (0, 2) |
| *Shigella* | 83 | 18.2 | (15, 22) | 21 | 5.8 | (4, 9) | 18 | 7.3 | (5, 11) |
| **ETEC** | 92 | 20.1 | (17, 24) | 46 | 12.6 | (10, 16) | 27 | 10.9 | (8, 15) |
| ***Giardia*** | 16 | 3.5 | (2, 6) | 12 | 3.3 | (2, 6) | 26 | 10.5 | (7, 15) |
| ***Cryptosporidium*** | 65 | 14.2 | (11, 18) | 43 | 11.8 | (9, 16) | 13 | 5.2 | (3, 9) |
|  | **n** | **median** | **(IQR)** | **n** | **median** | **(IQR)** | **N** | **median** | **(IQR)** |
| **Myeloperoxidase (ng/ml)** | 407 | 2.2 | (0.7, 5.6) | 320 | 1.2 | (0.5, 2.7) | 248 | 2.3 | (1.1, 5.4) |
| **Calprotectin (ug/ml)** | 383 | 194.2 | (70.9, 744.3) | 313 | 167.9 | (68.1, 393.7) | 242 | 252.4 | (124.0, 690.8) |
| **α-1-antitrypsin (mg/l)** | 406 | 85.7 | (33.3, 183.5) | 318 | 110.4 | (55.4, 224.7) | 247 | 201.3 | (100.2, 412.1) |

Abbreviations: EAEC: enteroaggregative *E. coli*, EPEC: enteropathogenic *E. coli*, ETEC: entero-toxigenic *E. coli*, IQR: interquartile range

**Table C. Pathogen prevalence among children without diarrhea at admission compared to the community children**

|  | **Admission** | | | **Discharge** | | | **Community** | | |
| --- | --- | --- | --- | --- | --- | --- | --- | --- | --- |
|  | **n** | **%** | **(95% CI)** | **n** | **%** | **(95% CI)** | **n** | **%** | **(95% CI)** |
| **Dehydrating^1^** | **42** | **11.9** | **(9, 16)** | **47** | **15.7** | **(12, 20)** | **42** | **11.9** | **(9, 16)** |
| Adenovirus | 15 | 4.2 | (3, 7) | 24 | 3 | (5, 12) | 15 | 4.2 | (3, 7) |
| Astrovirus | 8 | 2.3 | (1, 4) | 7 | 2.3 | (1, 5) | 2 | 0.8 | (0, 3) |
| Norovirus | 19 | 5.4 | (3, 8) | 24 | 8 | (5, 12) | 10 | 4.0 | (2, 7) |
| Rotavirus | 5 | 1.4 | (1, 3) | 10 | 3.3 | (2, 6) | 1 | 0.4 | (0, 2) |
| **Invasive^1^** | **247** | **69.8** | **(65, 74)** | **145** | **48.5** | **(43, 54)** | **151** | **60.9** | **(55, 67)** |
| *Aeromonas* | 2 | 0.6 | (0, 2) | 0 | 0 | (0, 1) | 3 | 1.2 | (0, 3) |
| *Campylobacter* | 90 | 25.4 | (21, 30) | 37 | 12.4 | (9, 17) | 49 | 19.8 | (15, 25) |
| EAEC | 189 | 53.4 | (48, 59) | 118 | 39.5 | (34, 45) | 99 | 39.9 | (34, 46) |
| Atypical EPEC | 41 | 11.6 | (9, 15) | 18 | 6 | (4, 9) | 22 | 8.9 | (6, 13) |
| Typical EPEC | 33 | 9.3 | (7, 13) | 10 | 3.3 | (2, 6) | 19 | 7.7 | (5, 12) |
| *Salmonella* | 7 | 2 | (1, 4) | 4 | 1.3 | (1, 3) | 1 | 0.4 | (0, 2) |
| *Shigella* | 17 | 4.8 | (3, 8) | 5 | 1.7 | (1, 4) | 18 | 7.3 | (5, 11) |
| **ETEC** | 50 | 14.1 | (11, 18) | 12 | 4 | (2, 7) | 27 | 10.9 | (8, 15) |
| ***Giardia*** | 40 | 11.3 | (8, 15) | 34 | 11.4 | (8, 15) | 26 | 10.5 | (7, 15) |
| ***Cryptosporidium*** | 29 | 8.2 | (6, 12) | 16 | 5.4 | (3, 9) | 13 | 5.2 | (3, 9) |
|  | **n** | **median** | **(IQR)** | **n** | **median** | **(IQR)** | **n** | **median** | **(IQR)** |
| **Myeloperoxidase (ng/ml)** | 274 | 3.3 | (1.3, 7.1) | 238 | 1.7 | (0.7, 4.2) | 248 | 2.3 | (1.1, 5.4) |
| **Calprotectin (ug/ml)** | 269 | 294.6 | (127.5, 993.1) | 236 | 173.0 | (81.8, 324.3) | 242 | 252.4 | (124.0, 690.8) |
| **α-1-antitrypsin (mg/l)** | 272 | 227.9 | (103.9, 502.0) | 237 | 177.8 | (109.0, 365.1) | 247 | 201.3 | (100.1, 412.1) |

Abbreviations: EAEC: enteroaggregative *E. coli*, EPEC: enteropathogenic *E. coli*, ETEC: entero-toxigenic *E. coli*, IQR: interquartile range

**Table D. Pathogen prevalence among children with both admission and discharge samples compared to the community children**

|  | **Admission** | | | **Discharge** | | | **Community** | | |
| --- | --- | --- | --- | --- | --- | --- | --- | --- | --- |
|  | **n** | **%** | **(95% CI)** | **n** | **%** | **(95% CI)** | **n** | **%** | **(95% CI)** |
| **Dehydrating^1^** | **163** | **29.2** | **(26, 33)** | **156** | **28.3** | **(25, 32)** | **20** | **8.1** | **(5, 12)** |
| Adenovirus | 30 | 0 | (4, 8) | 9 | 3.3 | (10, 16) | 7 | 0.8 | (1, 6) |
| Astrovirus | 19 | 3.4 | (2, 5) | 46 | 8.3 | 19 | 2 | 0.8 | (0, 3) |
| Norovirus | 40 | 7.2 | (5, 10) | 72 | 13 | 40 | 10 | 4.0 | (2, 7) |
| Rotavirus | 92 | 16.5 | (14, 20) | 49 | 8.9 | 92 | 1 | 0.4 | (0, 2) |
| **Invasive^1^** | **388** | **69.5** | **(66, 73)** | **335** | **60.7** | **(57, 65)** | **151** | **60.9** | **(55, 67)** |
| *Aeromonas* | 3 | 0.5 | (0, 2) | 2 | 0.4 | (0, 1) | 3 | 1.2 | (0, 3) |
| *Campylobacter* | 142 | 25.4 | (22, 29) | 76 | 13.8 | (11, 17) | 49 | 19.8 | (15, 25) |
| EAEC | 295 | 52.9 | (49, 57) | 285 | 51.6 | (47, 56) | 99 | 39.9 | (34, 46) |
| Atypical EPEC | 57 | 10.2 | (8, 13) | 33 | 6 | (4, 8) | 22 | 8.9 | (6, 13) |
| Typical EPEC | 76 | 13.6 | (11, 17) | 44 | 8 | (6, 11) | 19 | 7.7 | (5, 12) |
| *Salmonella* | 7 | 1.3 | (1, 3) | 7 | 1.3 | (1, 3) | 1 | 0.4 | (0, 2) |
| *Shigella* | 61 | 10.9 | (9, 14) | 21 | 3.8 | (3, 6) | 18 | 7.3 | (5, 11) |
| **ETEC** | 99 | 17.7 | (15, 21) | 54 | 9.8 | (8, 13) | 27 | 10.9 | (8, 15) |
| ***Giardia*** | 34 | 6.1 | (4, 8) | 35 | 6.3 | (5, 9) | 26 | 10.5 | (7, 15) |
| ***Cryptosporidium*** | 54 | 9.7 | (7, 12) | 50 | 9.1 | (7, 12) | 13 | 5.2 | (3, 9) |
|  | **n** | **median** | **(IQR)** | **n** | **median** | **(IQR)** | **n** | **median** | **(IQR)** |
| **Myeloperoxidase (ng/ml)** | 430 | 2.1 | (0.8, 5.3) | 558 | 1.413 | (0.6, 3.3) | 430 | 2.1 | (0.8, 5.3) |
| **Calprotectin (ug/ml)** | 408 | 197.9 | (73.4, 678.5) | 549 | 171.2 | (70.4, 369.7) | 408 | 197.9 | (73.4, 678.5) |
| **α-1-antitrypsin (mg/l)** | 428 | 110.2 | (44.2, 258.5) | 555 | 143.6 | (69.2, 278.6) | 428 | 110.2 | (44.2, 258.5) |

^1^Based on Kosek et al (2017), EAEC: enteroaggregative *E. coli*, EPEC: enteropathogenic *E. coli*, ETEC: enter-toxigenic *E. coli*, IQR: inter-quartile range

**Table E. Crude community correlates of ED biomarkers**

Note: No community children had oedema and children with diarrhea, pneumonia and sepsis were excluded from the community group.

|  | **Myeloperoxidase** | | **Calprotectin** | | **α-1-antitrypsin** | |
| --- | --- | --- | --- | --- | --- | --- |
|  | **Unadjusted** | | **Unadjusted** | | **Unadjusted** | |
|  | **Coef** | **(95% CI)** | **Coef** | **(95% CI)** | **Coef** | **(95% CI)** |
| MUAC | -0.05 | (-0.13, 0.03) | -0.03 | (-0.14, 0.08) | 0.01 | (-0.08, 0.11) |
| Height-for-age | 0.01 | (-0.06, 0.08) | 0.00 | (-0.09, 0.10) | 0.02 | (-0.06, 0.10) |
| Breastfeeding |  |  |  |  |  |  |
| Partial vs exclusive | -0.02 | (-0.31, 0.26) | 0.25 | (-0.14, 0.63) | -0.38 | (-0.72, -0.04) |
| None vs exclusive | **-0.38** | **(-0.72, -0.05)** | -0.05 | (-0.50, 0.40) | **-0.68** | **(-1.08, -0.28)** |
| Months of age |  |  |  |  |  |  |
| <6 vs >12 | **0.28** | **(0.08, 0.48)** | 0.23 | (-0.05, 0.51) | 0.07 | (-0.18, 0.32) |
| 6-12 vs >12 | 0.17 | (-0.09, 0.43) | -0.01 | (-0.36, 0.35) | 0.28 | (-0.04, 0.59) |
| Diagnosis malaria^1^ | 0.44 | (-0.37, 0.31) | 0.37 | (-1.59, 2.33) | -0.32 | (-2.06, 1.42) |
| Dehydrating pathogen | -0.03 | (-0.37, 0.31) | **0.51** | **(0.05, 0.96)** | 0.19 | (-0.22, 0.60) |
| Invasive pathogen | 0.17 | (-0.02, 0.36) | 0.24 | (-0.02, 0.49) | 0.09 | (-0.14, 0.32) |

^1^Based on positive malaria rapid diagnostic. MUAC: mid upper arm circumference.

**Table F. Association between individual pathogens and ED biomarkers at admission**

|  | **Myeloperoxidase** | | | **Calprotectin** | | | **α-1-antitrypsin** | | |
| --- | --- | --- | --- | --- | --- | --- | --- | --- | --- |
|  | **Coef** | **(95% CI,** | **p)** | **Coef** | **(95% CI,** | **p)** | **Coef** | **(95% CI,** | **p)** |
| **Dehydrating** |  |  |  |  |  |  |  |  |  |
| Adenovirus | 0.00 | (-0.02, 0.02, | 0.987) | 0.016 | (-0.00, 0.04, | 0.153) | 0.019 | (-0.00, 0.04, | 0.314) |
| Astrovirus | -0.08 | (-0.59, 0.42, | 0.746) | -0.15 | (-0.63, 0.34, | 0.555) | -0.25 | (-0.74, 0.24, | 0.314) |
| Norovirus | -0.25 | (-0.58, 0.08, | 0.134) | -0.06 | (-0.38, 0.26, | 0.716) | -0.02 | (-0.34, 0.30, | 0.886) |
| Rotavirus | -0.46 | (-0.69, -0.24, | 0.000) | -0.32 | (-0.55, -0.10, | 0.005) | -0.56 | (-0.77, -0.34, | 0.000) |
| **Invasive** |  |  |  |  |  |  |  |  |  |
| Aeromonas | 0.04 | (-0.10, 0.18, | 0.573) | 0.03 | (-0.10, 0.15, | 0.703) | -0.01 | (-0.14, 0.12, | 0.885) |
| *Campylobacter* | 0.12 | (-0.07, 0.30, | 0.211) | 0.10 | (-0.07, 0.28, | 0.249) | 0.06 | (-0.12, 0.23, | 0.546) |
| EAEC | 0.18 | (0.02, 0.34, | 0.031) | 0.20 | (0.05, 0.36, | 0.011) | -0.02 | (-0.18, 0.13, | 0.768) |
| Atypical EPEC | 0.05 | (-0.24, 0.33, | 0.749) | -0.05 | (-0.31, 0.22, | 0.742) | 0.03 | (-0.24, 0.30, | 0.842) |
| Typical EPEC | 0.17 | (-0.07, 0.40, | 0.160) | 0.01 | (-0.21, 0.23, | 0.918) | -0.12 | (-0.34, 0.10, | 0.293) |
| *Salmonella* | 0.06 | (-0.62, 0.74, | 0.862) | -0.17 | (-0.80, 0.46, | 0.588) | 0.28 | (-0.37, 0.93, | 0.396) |
| *Shigella* | 0.77 | (0.54, 1.01, | 0.000) | 0.60 | (0.38, 0.83, | 0.000) | -0.13 | (-0.37, 0.10, | 0.260) |
| **ETEC** | 0.06 | (-0.15, 0.28, | 0.566) | 0.01 | (-0.18, 0.22, | 0.853) | -0.04 | (-0.24, 0.17, | 0.709) |
| ***Giardia*** | 0.01 | (-0.31, 0.33, | 0.939) | 0.14 | (-0.17, 0.44, | 0.380) | 0.12 | (-0.19, 0.43, | 0.450) |
| ***Cryptosporidium*** | 0.05 | (-0.20, 0.31, | 0.674) | 0.04 | (-0.20, 0.28, | 0.730) | -0.15 | (-0.39, 0.10, | 0.242) |
