# Supplementary material for: Enteric dysfunction and enteropathogens among hospitalized south Asian and sub-Saharan African children
Source: PLOS Glob Public Health. 2026 Jul 24;6(7):e0006873. doi: 10.1371/journal.pgph.0006873 (PMC13399345; doi:10.1371/journal.pgph.0006873)
Supplement: Attachment — Submitted filename: Response letter_12Nov2025.pdf [file pgph.0006873.s004.pdf]

Editor-in-Chief

PLoS Global Public Health

November 14, 2025

Dear Editors,

Thank you for considering our appeal and offering us the opportunity to correct our incomplete submission of ethical documents. We have collected all the renewal forms for all the ethical review boards and uploaded them as .zip file. Please do tell us if you have challenges with this file format. The file includes:

OXTREC (UK) - initial letter confirming 5-years of approval that covers the study dates.

KEMRI (Kenya) - initial letter confirming 1-year of approval, and then subsequent renewals covering the enrollment period.

ICDDR,B (Bangladesh) - initial letter confirming 1-year of approval, and then subsequent renewals covering the enrollment period

AKU (Pakistan) - initial letter confirming 1-year of approval, and then subsequent renewals covering the enrollment period

Dow (Pakistan) - initial letter confirming 1-year of approval. Please note that when our team at AKU tried to renew the approval with the Dow University ethical review board we were informed that they had no extension system in place and this letter will suffice. If the Editors wish, please confirm this the leads of the AKU team, Dr. Ali Saleem (ali.saleem@aku.edu) and Ms Zaubina Kazi (zaubina.kazi@aku.edu).

COMREC (Malawi) - initial letter confirming 1-year of approval, and then subsequent renewals covering the enrollment period.

The Centre Muraz (Burkina Faso) - initial letter confirming approval for protocol, and the English translation.

Makerere (Uganda) - initial letter confirming 1-year of approval, and then subsequent renewals covering the enrollment period.

Uganda National Council for Science and Technology – intial letter covering the first three years, and an extension document.

OHSU (USA) – The initial determination from OHSU ethical review board stating that OHSU is not involved in human subjects research under this grant. Because it is unclear if this represents an approval, we have removed this ethical approval from the manuscript.

Sick Kids (Canada) - initial letter confirming 1-year of approval, and then subsequent renewals covering the enrollment period.

Please note, we have removed the reference to the University of Amsterdam providing ethical approval as the investigator from this site was not able to provide the relevant documents having left that institution. They were not involved in the human subjects research, but were a co-investigator working with the Malawi team. Therefore, we do not believe that the removal of the University of Amsterdam effects the ethical standings of the

Global WACh
Woman · Adolescent · Child · health

manuscript. We hope that this meets the ethical standards of the journal. As ever, we would be happy to make any further changes the editors advise.

Faithfully,

Dr K.D. Tickell

kirkbt@uw.edu

Department of Global Health, University of Washington, Seattle, USA.
