# Supplementary material for: Enteric dysfunction and enteropathogens among hospitalized south Asian and sub-Saharan African children
Source: PLOS Glob Public Health. 2026 Jul 24;6(7):e0006873. doi: 10.1371/journal.pgph.0006873 (PMC13399345; doi:10.1371/journal.pgph.0006873)
Supplement: Attachment — Submitted filename: Review.pdf [file pgph.0006873.s003.pdf]

Overall Comments:

● **What are the noteworthy results?**
The noteworthy results of this paper are that ED biomarkers were higher in hospitalized children at admission than in community children, and that by the time hospitalized children were discharged, their ED biomarkers were lower, sometimes even lower than community children. Additionally, this paper found that ED biomarkers were higher in children who had an invasive enteropathogen detected from a rectal swab. Finally, fecal calprotectin at discharge was associated with mortality.
● **Will the work be of significance to the field and related fields? How does it compare to the established literature? If the work is not original, please provide relevant references.**
This study strengthens the existing evidence for a potential connection between enteric dysfunction, enteric pathogens, and mortality in hospitalized children, finding consistently that sicker children have higher ED biomarkers and more enteric pathogens, that these markers of illness improve during their hospital stay, and that discharge levels of calprotectin were associated with mortality. This study improves upon the existing literature with a larger and more diverse cohort of children.
● **Does the work support the conclusions and claims, or is additional evidence needed?**
Yes, the work supports the conclusions and claims.
● **Are there any flaws in the data analysis, interpretation and conclusions? Do these prohibit publication or require revision?**
Yes, the statistical analysis lacks adjustment for multiple comparisons. Given the magnitude of comparisons made, this should be addressed. At the very least, the choice not to make such adjustments must be defended in the text.
● **Is the methodology sound? Does the work meet the expected standards in your field?**
Besides the lack of adjustment for multiple comparisons, the methodology is sound.
● **Is there enough detail provided in the methods for the work to be reproduced?**
Yes

Major Comments:

Introduction
Page 4, Line 80: Additional context regarding the causes of post-discharge mortality would be useful. I.e. manner of death, associated dehydration, diarrhea, cardiovascular problems etc.

Page 4, Line 84: A more nuanced treatment of the interrelated conditions listed here (EED, ED, malnutrition, GI infections, systemic inflammation, and microbiome dysbiosis) is warranted. Additionally, this sentence lacks proper citation or rationale.
Page 4, Line 92-97: A more thorough review of other studies examining ED biomarkers in hospitalized children in LMICs is warranted here. There are a few I do not see cited in the paper, particularly Sturgeon 2024 and the recent Allen 2025 in Nature Comms. Going over their findings and highlighting potential differences in the authors' study design would be a valuable addition. As this study is described in lines 94-97, be sure to highlight the timing of biomarker measurement (admission vs. discharge) and comparisons with community children.
Methods: Identification and adjustment of confounders was unclear in this section, though tables were helpful; please clarify and emphasize this part of the analysis. Additionally, please provide the coefficient of variation for the ELISA assays.

Discussion
Page 14, Line 327-329: I certainly would understand if the authors choose to address this in another work, but I would be very curious to see what additional analyses of the relationship between antibiotic use, ED biomarkers, and enteropathogen presence would reveal. For this paper, I wonder whether antibiotic use should have been run in the ED biomarker correlate analysis or as a potential confounder in every multivariate analysis performed. It would be interesting to see whether antibiotics would be associated with more detection of norovirus, for example.
Page 14, Line 329–331: Including some additional information and citations regarding the impacts of sanitation changes on ED biomarkers and enteropathogens would strengthen this hypothesis.
Page 15, Line 338-341: This idea would be strengthened if the authors would add information about the details of post-discharge morbidity and mortality here or elsewhere in the introduction/discussion.
Page 15, Line 346: Additional information about budesonide, as the authors have provided about teduglutide and mesalazine, is warranted.
Table 1: Would be valuable to add reason for hospitalization to the characteristics table, or somewhere else if more appropriate, assuming this would be different than Diagnoses. If diagnoses is equivalent to reason for hospitalization please clarify this.
Figure 2: Resolution is poor on this image. Include n for each group. Note that most papers use ng/mL to report MPO concentrations (see Kosek 2013). Keeping units the same from paper to paper is important for the purpose of comparison. It also appears the authors use different units in tables vs. this figure, please amend. This image tells a strong story that did not come off so clearly in the text of the results - perhaps consider strengthening the text for this section.

S2 Table 6: This table should be included in the main text given that it is a whole section of the results.

Minor Comments:

Page 3, Line 65: strike “was associated with mortality”; redundant with first part of sentence.
Page 4, Line 82: add microbial to specify what kind of translocation
Page 4, Line 92: would be valuable to add some additional citations after enteroinvasive to further support the consensus on the relationship between ED biomarkers and enteropathogens.
Page 10, Line 229: Awk sentence, recommend adding a semicolon after “During hospitalization norovirus presences nearly doubled”
Page 14, Lines 313-316: Recommend reordering these two sentences so that the statement outlining how the authors’ evidence supports the hypothesis concludes the paragraph.
Page 14, Line 325: typo; change “his” to “this”
Page 15, Line 349: Formatting may have been altered by forces outside the authors’ control but be sure to italize latin name for *P. falciparum* malaria.
Page 17, Line 381: typo; change “diagnostic is” to “diagnostics are”
Table 3: Appears to be a missed bold or typo in None vs. exclusive breastfeeding; a-1-antitrypsin for adjusted comparison; Coef is positive, 95% CI is negative. Typo in second line under table. Change “due” to “of”. Appears to be minor alignment issue with center column as coef’s are lower than corresponding CIs.
