# Supplementary material for: Enteric dysfunction and enteropathogens among hospitalized south Asian and sub-Saharan African children
Source: PLOS Glob Public Health. 2026 Jul 24;6(7):e0006873. doi: 10.1371/journal.pgph.0006873 (PMC13399345; doi:10.1371/journal.pgph.0006873)
Supplement: Attachment — Submitted filename: Reviewer_comment_response_05May_2026.docx [file pgph.0006873.s005.docx]

**Reviewer #1**: Overall Comments:


**Major Comments:**

Introduction
Page 4, Line 80: Additional context regarding the causes of post-discharge mortality would be useful. I.e. manner of death, associated dehydration, diarrhea, cardiovascular problems etc.

**Thank you for suggesting this addition. We are not able to provide a comprehensive overview of post-discharge mortality aetiology as we do not believe one exists. Many deaths occur in the community with unknown causes. Within the CHAIN study an expert panel did attribute causes of post-discharge death as far as possible. However, we believe fairest assessment of aetiology is that the causes of post-discharge mortality are as broad as those of inpatient deaths. Consequently, we have added the following statement to the manuscript:**

***L80-84: “Many of these post-discharge deaths occur within the first 45 days following hospitalization, and are attributable to broad range of acute and chronic conditions. However, the risk of mortality remains elevated up to six or even 12 months after hospitalization suggesting that underlying vulnerabilities predispose these children to poor outcomes.”***

Page 4, Line 84: A more nuanced treatment of the interrelated conditions listed here (EED, ED, malnutrition, GI infections, systemic inflammation, and microbiome dysbiosis) is warranted. Additionally, this sentence lacks proper citation or rationale.

**We have made the following revisions:**

***L86-103: “Enteric dysfunction (ED) due to various etiologies may contribute to poor outcomes after hospital discharge. Environmental enteric dysfunction (EED) is a prevalent, largely asymptomatic condition in LMICs characterized by small bowel injury, inflammation, increased gut permeability, translocation of microbes or microbial products, and systemic inflammation. EED is highly consequential to child growth and neurodevelopment, with impacts on health across the life course. Frequent exposure to enteric pathogens has been associated with EED among children in the community. Children admitted to hospital have a high prevalence of other conditions which can also cause ED, including malnutrition, acute gastrointestinal infections, and chronic infections such as HIV. Furthermore, exposure to treatments, such as antibiotics and therapeutic foods, can disrupt the gut microbiome and intestinal homeostasis. Data from Zambia and Zimbabwe associated ED and systemic inflammation biomarkers with increased risk of mortality and rehospitalization in the post-discharge period among children with severe acute malnutrition. Data from the cohort used for this analysis found detection lipopolysaccharide, a marker of translocation of gram negative bacteria or bacterial antigens, to be associated with mortality among hospitalized children. A deeper understanding of ED, broadly defined and regardless of specific etiology, during acute illness may lead to interventions that prevent post-discharge morbidity and mortality***.***”***

Page 4, Line 92-97: A more thorough review of other studies examining ED biomarkers in hospitalized children in LMICs is warranted here. There are a few I do not see cited in the paper, particularly Sturgeon 2024 and the recent Allen 2025 in Nature Comms. Going over their findings and highlighting potential differences in the authors’ study design would be a valuable addition. As this study is described in lines 94-97, be sure to highlight the timing of biomarker measurement (admission vs. discharge) and comparisons with community children.

**We have added discussion of these studies to the introduction to help frame the central hypotheses of this manuscript.**

***L91103: “Children admitted to hospital have a high prevalence of other conditions which can also cause ED, including malnutrition, acute gastrointestinal infections, and chronic infections such as HIV. Furthermore, exposure to treatments, such as antibiotics and therapeutic foods, can disrupt the gut microbiome and intestinal homeostasis. Data from Zambia and Zimbabwe associated ED and systemic inflammation biomarkers with increased risk of mortality and rehospitalization in the post-discharge period among children with severe acute malnutrition. Data from the cohort used for this analysis found detection lipopolysaccharide, a marker of translocation of gram negative bacteria or bacterial antigens, to be associated with mortality among hospitalized children. A deeper understanding of ED, broadly defined and regardless of specific etiology, during acute illness may lead to interventions that prevent post-discharge morbidity and mortality***.***”***

Methods: Identification and adjustment of confounders was unclear in this section, though tables were helpful; please clarify and emphasize this part of the analysis. Additionally, please provide the coefficient of variation for the ELISA assays.

**Thank you for this important feedback, the coefficients of variation have been added, and the following clarifications have been made regarding confounders:**

***L224-228: “These models were then adjusted for admission variables a-priori identified confounder from the correlates analysis above (age, sex, MUAC, and enrollment site), in addition to other important risk factors for ED and mortality including LAZ, current breastfeeding (none, partial, exclusive), HIV status (infected/uninfected), invasive pathogen detection, caregiver reported recent diarrhea, and diagnoses of pneumonia, malaria and sepsis.”***

***L232-236: “Confounder adjusted post-discharge models included age, sex, discharge MUAC, enrollment site, discharge LAZ, current breastfeeding (none, partial, exclusive), HIV status (infected/uninfected), enteroinvasive pathogen detection at discharge, reported diarrhea at admission, and diagnoses of pneumonia, malaria and sepsis during admission. All analyses were performed in R (v3.6.1) with p<0.05 (two-tailed) considered significant.”***

Discussion
Page 14, Line 327-329: I certainly would understand if the authors choose to address this in another work, but I would be very curious to see what additional analyses of the relationship between antibiotic use, ED biomarkers, and enteropathogen presence would reveal. For this paper, I wonder whether antibiotic use should have been run in the ED biomarker correlate analysis or as a potential confounder in every multivariate analysis performed. It would be interesting to see whether antibiotics would be associated with more detection of norovirus, for example.

**This is a really interesting question. We had not thought about the potential link between antibiotic use and norovirus acquisition, and we agree that there should be a link between antibiotic use and bacterial pathogens. Two key challenges of the proposed analyses are 1) almost all the children were given antibiotics during their admission, and 2) there would be strong confounding by indication in this analysis. Due to these challenges, we believe that answering these questions warrants a separate carefully considered analysis and interpretation. Therefore, as the reviewer suggested, we will leave this analysis for a future project.**

Page 14, Line 329–331: Including some additional information and citations regarding the impacts of sanitation changes on ED biomarkers and enteropathogens would strengthen this hypothesis.

**We have added this to the discussion in this revision: *L361-366 “Changes in water and sanitation exposures and food types and preparation practices may also have contributed to declines in enteropathogens during hospitalization, although the evidence for the effectiveness of these interventions in community setting is mixed.”***

Page 15, Line 338-341: This idea would be strengthened if the authors would add information about the details of post-discharge morbidity and mortality here or elsewhere in the introduction/discussion.

**We have added additional information to the introduction and in response to this comment we have added to the discussion:**

***Introduction L78-84: “Pediatric mortality following discharge from hospitals in low- and middle-income countries (LMICs) is unacceptably high, with estimates suggesting that nearly half of deaths among hospitalized children occurring post-discharge. Many of these post-discharge deaths occur within the first 45 days following hospitalization, and are attributable to broad range of acute and chronic conditions. However, the risk of mortality remains elevated up to six or even 12 months after hospitalization suggesting that underlying vulnerabilities predispose these children to poor outcomes.”***

***L 371-376: “Both systemic inflammation and poor nutritional status have been shown to be strongly predictors of post-discharge mortality. It is possible that post-discharge morbidity and mortality could be reduced by therapeutics that help children avoid re-colonization with enteroinvasive pathogens or extend the period of reduced enteric inflammation after hospitalization by promoting nutrient uptake and decreasing systemic inflammation.”***

Page 15, Line 346: Additional information about budesonide, as the authors have provided about teduglutide and mesalazine, is warranted.

**We have added the following information:**

***Line 378-382: “A recent trial of enteric interventions at hospital discharge among children with severe acute malnutrition found that teduglutide, a glucagon-like peptide that promotes mucosal regeneration, reduced enteric inflammation, while oral budesonide, a corticosteroid active in the gut, was associated with reduced plasma*** ***C-reactive protein.”***

Table 1: Would be valuable to add reason for hospitalization to the characteristics table, or somewhere else if more appropriate, assuming this would be different than Diagnoses. If diagnoses is equivalent to reason for hospitalization please clarify this.

**We have added the presenting complaints to the Table 1.**

Figure 2: Resolution is poor on this image. Include n for each group. Note that most papers use ng/mL to report MPO concentrations (see Kosek 2013). Keeping units the same from paper to paper is important for the purpose of comparison. It also appears the authors use different units in tables vs. this figure, please amend. This image tells a strong story that did not come off so clearly in the text of the results - perhaps consider strengthening the text for this section.

**As recommended, we have standardized the units to ng/ml across the figure, tables and text and the resolution of the text has been improved. We note that the figure as displayed in the system generated PDF has poor resolution, but if the our upload is downloaded direct the resolution is good.**

S2 Table 6: This table should be included in the main text given that it is a whole section of the results.

**We have moved this table to the main results as per your recommendation.**

**Minor Comments:**

Page 3, Line 65: strike “was associated with mortality”; redundant with first part of sentence.

**Thank you – this has been corrected.**

Page 4, Line 82: add microbial to specify what kind of translocation

**We have added “microbial or microbial products ” (Line 89).**

Page 4, Line 92: would be valuable to add some additional citations after enteroinvasive to further support the consensus on the relationship between ED biomarkers and enteropathogens.

**This section of the introduction has been extensively revised and the citations have been strengthened.**

Page 10, Line 229: Awk sentence, recommend adding a semicolon after “During hospitalization norovirus presences nearly doubled”

**This sentence has been revised to:**

**L254-256: *“During hospitalization norovirus prevalence nearly doubled (p<0.001), with no sites observing a decrease in norovirus detection, and seven sites having a higher prevalence at discharge than admission.”***

Page 14, Lines 313-316: Recommend reordering these two sentences so that the statement outlining how the authors’ evidence supports the hypothesis concludes the paragraph.

**Thank you – this has been corrected (L345).**

***“Biomarkers of enteric inflammation, including fecal myeloperoxidase and calprotectin, decreased during hospitalization to levels significantly lower than community peers, although the majority of children at admission, discharge (and in the community) had levels above what is considered normal for age (2000ng/ml for myeloperoxidase and 77 ug/ml for calprotectin).*** ***We also observed a decrease in invasive enteropathogen prevalence during hospitalization and the detection of invasive enteropathogens was strongly associated with enteric inflammation biomarkers among hospitalized children. Finally, fecal calprotectin at discharge from hospital was associated with post-discharge mortality in the adjusted model. Collectively, these data support the hypothesis that a high prevalence of invasive enteropathogens promotes enteric inflammation among acutely unwell children.”***

Page 14, Line 325: typo; change “his” to “this”

**Thank you – this has been corrected.**

Page 15, Line 349: Formatting may have been altered by forces outside the authors’ control but be sure to italize latin name for P. falciparum malaria.

**Thank you for giving us the benefit of the doubt, and this has now been corrected.**

Page 17, Line 381: typo; change “diagnostic is” to “diagnostics are”

**Thank you – this has been corrected.**

Table 3: Appears to be a missed bold or typo in None vs. exclusive breastfeeding; a-1-antitrypsin for adjusted comparison; Coef is positive, 95% CI is negative. Typo in second line under table. Change “due” to “of”. Appears to be minor alignment issue with center column as coef’s are lower than corresponding CIs.

**Thank you – these errors have been corrected.**

**Reviewer #2**: This manuscript addresses an important and underexplored question regarding enteric dysfunction and enteropathogen burden in hospitalized children across LMICs. The data are drawn from the well-established CHAIN cohort and benefit from a large, multi-site, multi-country design. However, the manuscript has several substantive methodological, analytical, and interpretive concerns that must be addressed before it can be considered for publication. The specific issues are detailed below, organized by section, with line numbers provided for the authors' reference.

**Thank you for your support, we have addressed the issues below.**

Abstract
Lines 48-49 vs. Lines 199-200: abstract states that cox-proportional hazard models assessed ED biomarker associations with 'inpatient and post-discharge mortality.' while methods later clarifies that 30-day period following admission was chosen. Why are these two framings contradictory? The abstract must be corrected to accurately reflect what was actually measured.

**That is very good point. We have corrected the abstract to state 30-day mortality.**

**L48-49: *“Cox-proportional hazard models assessed ED biomarker associations with mortality in the 30-days following admission, and 180-days following discharge”***

Lines 53-55: Calprotectin at discharge was significantly lower than community, yet the abstract goes on to report that discharge calprotectin was the only biomarker associated with mortality. If discharge calprotectin was not elevated, the premise that enteric inflammation at admission is a clinically important finding in this hospitalized group becomes difficult to defend. The authors need to address why a biomarker that behaves no differently from the community would carry mortality-predictive value at discharge.

**We find this to be a very interesting observation on our data that we had not previously considered. We have added the reviewer’s observation to the discussion with our interpretation of its meaning.**

**L411-419: *“We did find that higher discharge calprotectin levels were associated with post-discharge mortality, potentially indicating that enteric inflammation undermines recovery in the post-discharge period. It is interesting to note that while the mean calprotectin was lower among children at admission, and especially at discharge, compared to community peers, the mean admission and discharge calprotectin concentrations were above levels considered within normal limits for age and even above levels considered sensitive and specific for inflammatory bowel disease. The association calprotectin-post-discharge mortality association suggests that enteric inflammation interplays with other factors among vulnerable children to increase the risk of death.”***

Introduction
Lines 78-97: The introduction is well-structured and provides limited background. However, it does not provide the specific hypothesis being tested regarding whether enteric dysfunction in acutely ill hospitalized children is etiologically distinct from environmental enteric dysfunction in community children. This distinction is critical for interpreting the results and should be stated more explicitly.

**We have clarified the introduction to better define the technical difference between ED and EED and to more clearly state the hypothesis as you have framed it.**

***L86-103: ““Enteric dysfunction (ED) due to various etiologies may contribute to poor outcomes after hospital discharge. Environmental enteric dysfunction (EED) is a prevalent, largely asymptomatic condition in LMICs characterized by small bowel injury, inflammation, increased gut permeability, translocation of microbes or microbial products, and systemic inflammation. EED is highly consequential to child growth and neurodevelopment, with impacts on health across the life course. Frequent exposure to enteric pathogens has been associated with EED among children in the community. Children admitted to hospital have a high prevalence of other conditions which can also cause ED, including malnutrition, acute gastrointestinal infections, and chronic infections such as HIV. Furthermore, exposure to treatments, such as antibiotics and therapeutic foods, can disrupt the gut microbiome and intestinal homeostasis. Data from Zambia and Zimbabwe associated ED and systemic inflammation biomarkers with increased risk of mortality and rehospitalization in the post-discharge period among children with severe acute malnutrition. Data from the cohort used for this analysis found detection lipopolysaccharide, a marker of translocation of gram negative bacteria or bacterial antigens, to be associated with mortality among hospitalized children. A deeper understanding of ED, broadly defined and regardless of specific etiology, during acute illness may lead to interventions that prevent post-discharge morbidity and mortality***.***”***

***L110-113: “This analysis aimed to understand a) trends in ED biomarkers and enteropathogens in acutely unwell children across a nutritional spectrum in LMICs, b) enteropathogen, clinical, and sociodemographic correlates of ED, and c) ED biomarkers’ association with mortality in the 30-days following admission and 180-days following discharge.”***

Methods
Lines 128-132: What is the justification for nested case-cohort (NCC) randomly selected 24% of children from the CHAIN cohort along with all deaths. Was a formal sample size calculation performed to ensure this sub-cohort was powered to detect associations between ED biomarkers and mortality? If so, the assumed effect sizes, alpha, and power should be reported. If not, this is a significant methodological gap that must be acknowledged.

**Details of the sample size calculation are offered in the overarching protocol paper (Njunge 2022). However, we have added further details to the current manuscript:**

**L144-149: *“The CHAIN nested case cohort (NCC) analyzed CHAIN samples to gain further insights into mortality, and was powered to detect a Hazard Ratio of 1.5 with 80% power in both the 30-day and 180-day mortality analyses.”***

Lines 129-132: The decision to recruit 30 community children per site also lacks justification.

**The community cohort is intended to provide normative levels for biomarkers that are not well understood in these communities. Generally, the minimum number of people required to get reliable estimate of mean biomarker concentrations is approximately 30. However, no specific power calculation was conducted to support that selection.**

***L148: “30 community children from each site (to provide reference values for biomarkers without well-established clinical cutoffs).”***

Lines 199-201: The authors state that the 30-day period following hospital admission was chosen because 'inpatient mortality is biased by discharge decisions.' While this reasoning is understandable, labelling this as inpatient mortality anywhere in the manuscript is inaccurate. Children discharged within that 30-day window who subsequently die are not inpatient deaths.

**We agree with the reviewer’s observation and we have removed any discussion of inpatient mortality.**

Lines 199-204: Using ED biomarkers measured at admission to predict mortality over the subsequent 30 days, without accounting for or even reporting the number of days each child remained hospitalized, is a fundamental flaw. A child admitted with a high myeloperoxidase value who dies on day two of admission versus from one who survives, is treated, and dies on day 28? The length of hospital stay is not reported anywhere in the manuscript, no mean or median duration of admission is provided despite the discussion (line 327) mentioning 'an average of five days.' If this figure exists, it belongs in Table 1 or the results section, and its implications for the mortality models must be examined.

**We have added the length of hospital to the results (L248), and report a median of 5 days (IQR 3-8 days, minimum 0 days and maximum 165). We found it hard to include this statistic in Table 1 as “Admission” and “Discharge” columns are essentially the same children, and the “Community” column were by definition not admitted. However, this information is now clearly stated in the results.**

**We agree with the reviewer’s intuition that early and late deaths are likely to be mechanistically very different, and also agree with this principle in the post-discharge analysis discussed lower in this response. However, we feel it is methodologically important for the mortality analysis to describe the whole period of mortality and to align with primary analysis of mortality in the CHAIN manuscript. As we hope that the biological investigations conducted in this manuscript, and other CHAIN papers, will help understand the broader trends observed in CHAIN’s and post-discharge mortality results. Additionally, we feel it would be hard to interpret admission biomarker’s associations with late mortality, when they are perhaps most likely to be relevant, by excluding early deaths. We believe this would introduce a very substantial bias into the mortality analysis, and would require a considered methodological approach and separate manuscript to report that analysis. Therefore, we have attempted to disaggregate mortality in this current analysis.**

Lines 206-211: Using discharge biomarker concentrations to predict mortality over 180 days following discharge is a very long causal window. The biological rationale for why a stool biomarker measured at the point of discharge would independently predict mortality six months later, against the backdrop of all the life events, re-infections, nutritional deterioration, or new illnesses that could occur in that period, is not adequately established. The authors must provide a scientific justification for this association as it is highlighted in the conclusions that is drawn from it.

**We agree with the reviewer that this is long window but, we hope that this analysis presents an overview of the mortality associations and replicated the mortality analysis of the parent cohort adding a biological assessment of EED. We feel it is important to start with this overarching view of mortality in the post-discharge period to ensure that any insights to gains can be related to the parent analysis which found elevated mortality for at least 6-months following discharge. It would be interesting secondary analysis to establish it any observed effects evolve over time during the post-discharge period, but this complex analysis would require considerable thought and would be deserving of its own manuscript. Therefore, we have chosen to not further disaggregate the post-discharge period.**

Lines 202-210: MUAC is used as a covariate in both the admission and discharge models. The authors should clarify whether admission MUAC or discharge MUAC is used in each respective model. More importantly, it is biologically implausible that MUAC would change meaningfully over a median hospital stay of five days in these children. If admission MUAC is used in the discharge model, this needs to be justified. If discharge MUAC is used, the authors need to explain what meaningful change is expected in this short a window in acutely ill malnourished children, and whether this creates collinearity with admission MUAC.

**The MUAC corresponding to the time point was used for each model (i.e. the discharge model uses discharge MUAC). The reviewer is correct that practically speaking the vast majority of MUACs will change little during a short hospitalization. However, some change can occur during rehydration (as per Mwangome et al Nutr J. 2011 Sep 13;10:92. doi:** [**10.1186/1475-2891-10-92**](https://doi.org/10.1186/1475-2891-10-92)**) and some children do experience longer hospitalization. However, we simply felt it was cleaner to use discharge MUAC in a discharge model. We have the revised the manuscript to clarify which MUAC was used at which time point.**

**L231-236: *“Confounder adjusted post-discharge models included age, sex, discharge MUAC, enrollment site, discharge LAZ, current breastfeeding (none, partial, exclusive), HIV status (infected/uninfected), enteroinvasive pathogen detection at discharge, reported diarrhea at admission, and diagnoses of pneumonia, malaria and sepsis during admission.”***

Results
Line 218: The statement that hospitalized children were 'less commonly exclusive breastfed' requires clarification. A comparison of breastfeeding rates across the broad age group confound the age-appropriate feeding practices with what may simply be an age distribution difference between the hospitalized and community groups. This should be stratified by age group or the comparison should be appropriately caveated.

**Thank you for highlighting this issue, we agree with the reviewer that the age of a child almost certainly influences the probability they breastfed and more so their need to exclusively breastfeed. As our community children were selected to mirror the ages of the hospitalized cohort, the raw percentages should be somewhat comparable across the groups. However, we have added the percentages among the infants under 6 months as a foot note to Table 1.**

Lines 216-222: Table 1 provides sociodemographic and clinical characteristics but does not report the distribution of MUAC strata (no wasting, moderate wasting, severe wasting or kwashiorkor) separately for the hospitalized group. Given that enrollment was deliberately stratified by MUAC in a 2-1-2 ratio, and that MUAC is strongly associated with both biomarker levels and mortality, the reader needs to know how many children fell into each nutritional stratum. Failure to present this renders the clinical context of the acutely ill cohort incomplete and makes it impossible to assess the degree of confounding by nutritional status.

**The original CHAIN study classification of wasting has been added to the Table 1 as per the reviewer’s recommendation.**

Lines 225-231: The paper compares the prevalence of dehydrating pathogens between hospitalized and community children and reports a significant difference (25-27% vs 8%, p<0.001). While this is numerically striking, the clinical relevance of this comparison is questionable. Children admitted to hospital are there precisely because they are acutely unwell, and a significant proportion presented with diarrhea. Comparing pathogen prevalence for dehydrating organisms in acutely ill hospitalized children with healthy community children tells us little beyond the expected. The authors should clarify what clinical or public health inference they intend the reader to draw from this comparison.

**The reviewer is correct, this is not intended to be a clinical impactful result, but is necessary to fulfil or obligation to report our results fully. We offer no further comment on this finding in the manuscript because it does not add to the existing literature. However, omission of this result would constitute incomplete reporting of our findings, therefore, we feel it must be retained.**

Lines 242-243: Giardia was lower among hospitalized children at both admission and discharge compared to community children (7% vs 11%), and this finding is never discussed. Given that Giardia is associated with asymptomatic carriage and positive growth outcomes in some cohorts, its higher prevalence in the community group warrants at least a brief acknowledgment in the discussion.

**We have added a sentence acknowledging this finding to the discussion.**

***L354-356: “Giardia appeared to be more common among community than hospitalized children, as has been observed in other settings, although the difference was not significant.”***

Lines 246-252: The alpha-1-antitrypsin findings are counterintuitive and insufficiently explained in the results. Concentrations are lower at admission compared to the community, but then recover to community levels by discharge. The direction of this change is opposite to that of the inflammatory markers and the biological interpretation is unclear. The authors invoke a dilutional effect of diarrhea in the discussion, but this is not examined rigorously in the results. Given that this biomarker reflects intestinal permeability, not inflammation, and given that these children are acutely ill with multiple conditions that could compromise gut barrier function, this unexpected pattern deserves a proper mechanistic discussion rather than a brief mentioning.

**We have added a further discussion:**

**L384-392: *“The observed increase in levels of fecal α-1-antitrypsin, an enteric permeability biomarker, during hospitalization was in the opposite direction compared to the enteric inflammatory biomarkers. However, this trend was isolated to children who had presented with diarrhea. A dilutional effect of diarrhea on α-1-antitrypsin concentrations at admission is highly likely, supported by our finding of a strong association between lower α-1-antitrypsin concentrations and dehydrating enteropathogens. However, α-1-antitrypsin did appear to be raised among children with either pneumonia or malaria. P. Falciparum malaria sequesters in intestinal capillaries and is thought to cause local hypoxia and increased permeability which may explain the association with α-1-antitrypsin.”***

Lines 254-256: The text states that 'trends in pathogen prevalence were similar among hospitalized children with and without reported diarrhea,' yet the very next sentence reports that children with diarrhea had higher rotavirus, norovirus, typical EPEC, and Shigella prevalence. These two statements are not consistent. If the distributions of individual pathogens differ substantially by diarrhea status, the overall trends cannot be described as similar without qualification.

**We have clarified: L281-285 *“*Children with reported diarrhea had higher rotavirus, norovirus, typical EPEC and Shigella prevalence at admission, but fewer detections of Giardia than children without diarrhea. However, the trend of decreased pathogen prevalence between admission and discharge, particularly invasive enteropathogens, was similar among hospitalized children with and without reported diarrhea (S2 Tables 1 & 2).”**

Lines 267-285 (Table 3): The correlate models in Table 3 are restricted to the hospitalized cohort. No equivalent model is presented for community children. It is therefore unknown whether the association between lower MUAC and higher myeloperoxidase or calprotectin is specific to the acutely ill or is equally present in the community. The same applies to the association with invasive enteropathogens. Without a community reference model, it is impossible to determine whether these associations are a feature of acute illness or simply reflect the biology of these children in their home environment.

**We have added a correlates of ED biomarkers to the supplement to allow for comparison. Many of clinical factors (e.g. pneumonia, diarrhea) were exclusion criteria among the community children. However, the association between the basic sociodemographic of these children and the ED biomarker concentrations have been given in the revised table (Table 3).**

Lines 267-285 (Table 3): Acutely ill children in this cohort have a high prevalence of sepsis (13.4%), pneumonia (39.6%), and malaria (12.6%). Fecal biomarkers such as myeloperoxidase and calprotectin reflect systemic as well as local intestinal inflammation. In children with these co-morbidities, attributing elevated biomarker values to enteropathogen detection, whether or not diarrhea is present, is methodologically problematic. The presence of these systemic illnesses is a major unmeasured confounder for the enteropathogen-biomarker relationship, and this is insufficiently acknowledged.

**We fully understand and agree with the reviewer’s concerns about drawing inference from this observational study and further cautioned readers about the risks of confounding in this study in our discussion. The section the reviewer references here is result section reporting the clinical correlates of biomarkers. We note that Table 3 is clearly labelled as a “correlates” table and the subsection of the results referenced by the reviewer is also described as “Correlates of ED biomarkers at admission. We have further reviewed this section of the results and believe it appropriately describes the key results listed in Table 3 without attempting inference. Therefore, we have limited the revisions made in response to this comment to the discussion.**

***L422 “However, the analysis has several limitations. These data were observational limiting our ability to infer causality. For example, systemic inflammation is known to be associated with post-discharge outcomes. While systemic inflammation is considered to be a downstream consequence of enteric inflammation, it is plausible that systemic inflammation may contribute to enteric inflammation. We did adjust for major diagnoses associated with systemic inflammation – namely sepsis, pneumonia, and malaria. However, systemic inflammation, especially due to other etiologies could be a residual confounder in the association between discharge calprotectin and mortality.”***

**We feel somewhat confident in our focus on enteric pathogens in this analysis partially because the main line of Ed or EED research hypothesizes that enteric pathogens are source of enteric inflammation and therefore our focus is in keeping with the rest of the field. Secondly, invasive enteric pathogens have strong association with enteric inflammation in our data, whereas malaria, pneumonia and sepsis do not. This suggest it is not plausible that these factors act as major confounders in our data as all confounders must be associated with the outcome. However, the reviewer is correct to caution against over interpretation of the data.**

Lines 293-297: The crude models found that admission myeloperoxidase and calprotectin were associated with 30-day mortality, but this association was lost after adjustment. This is an important null finding that deserves more interpretive weight in the discussion. If the biomarker-mortality relationship at admission disappears entirely after adjusting for MUAC, age, and co-morbidities, then the biomarkers add no independent prognostic information at admission. The authors should reflect on what this means for the utility of measuring these biomarkers at admission?

**We have added further interpretation to the discussion of this finding.**

***L402-409: “The associations between ED biomarkers at admission and mortality were strongly confounded by factors such as MUAC, age and background illnesses suggesting ED at admission is either not a driver of mortality, that its contribution is masked by other factors of more substantial magnitude, or that it acts as a mediator for known risks factors of mortality.”***

Lines 299-304: The finding that discharge calprotectin is associated with 180-day post-discharge mortality, while no admission biomarker retains significance after adjustment, raises the question of whether this reflects a true biological relationship or residual confounding. Discharge calprotectin was already lower than community levels (171.2 vs 252.4 ug/ml). Explaining how a biomarker that is already below community norms at discharge independently predicts mortality six months later requires more than a hazard ratio; it requires a mechanistic and contextual argument that is not currently present.

**As noted above, we find this to be a very interesting observation on our data that we had not previously considered. We have added the reviewer’s observation to the discussion with our interpretation of its meaning:**

**L411-419: *“******We did find that higher discharge calprotectin levels were associated with post-discharge mortality, potentially indicating that enteric inflammation undermines recovery in the post-discharge period. It is interesting to note that while the mean calprotectin was lower among children at admission, and especially at discharge, compared to community peers, the mean admission and discharge calprotectin concentrations were above levels considered within normal limits for age and even above levels considered sensitive and specific for inflammatory bowel disease. The association calprotectin-post-discharge mortality association suggests that enteric inflammation interplays with other factors among vulnerable children to increase the risk of death.”***

Lines 299-304: The text does not clearly specify for which model admission versus discharge biomarker values were used. The description of crude and adjusted models for 30-day and 180-day outcomes would benefit from explicit clarification in each case as to which timepoint's biomarker was entered into each model.

**This section has been revised for clarity:**

**L327-332: *“Myeloperoxidase and calprotectin at discharge were associated with 180-day mortality following discharge in crude models. Discharge myeloperoxidase was not associated with mortality after adjustment, but a one SD increase in calprotectin at discharge was associated with 36% (hazard ratio 1.36, 95% CI: 1.07,1.72, p=0.013) mortality increase. α-1-antitrypsin at discharge was not associated with 180-day post-discharge mortality in either crude or adjusted models.”***

Lines 299-304: The HIV-infected rate in this cohort is 6.7%. HIV infection is independently associated with enteropathy, impaired immunity, and mortality. The handling of HIV-infected children in the mortality models is not described. Were they excluded, included without stratification, or included with HIV status as a covariate? Given the known impact of HIV on both enteric inflammation and post-discharge mortality, this requires explicit reporting.

**This is a very good point. We have now included HIV as an adjustment factor in all the mortality models. It does not substantively change our findings.**

Figure 2
Figure 2: A dedicated figure is presented comparing myeloperoxidase at admission and discharge, stratified by invasive pathogen detection. However, myeloperoxidase was not associated with mortality in either crude or adjusted post-discharge models. Presenting a detailed figure for a biomarker that does not ultimately predict the primary outcome of interest, while calprotectin, which did retain significance, is not similarly visualized, represents a disconnect between the analytical focus and the visual emphasis of the manuscript. The authors should reconsider this figure or add a parallel figure for calprotectin.

**Thank you for your considered comment here, as requested we have added a similar figure for calprotectin as a supplementary appendix. While we do hope that this discovery research will contribute to reduce post-discharge mortality, we aimed in this project to describe high level trends in these biomarkers and therefore associations. As such we believe this figure describes two important trends observed in the data 1) the decrease from admission to discharge, 2) the association with detection of invasive pathogens. We feel it is helpful to the readers to retain this figure in the manuscript, although the reviewer is correct that the addition of a similar figure for calprotectin will also help readers.**

Discussion
Lines 307-397: The discussion section reads largely as an overview of the broader literature on enteric dysfunction and EED rather than an interpretation of the authors' own findings. Key results from this study, particularly the null findings for myeloperoxidase and alpha-1-antitrypsin in the mortality models, the counterintuitive direction of alpha-1-antitrypsin across timepoints, and the paradox of below-community calprotectin at discharge predicting mortality six months later, are not given adequate analytical attention. The discussion should be restructured to lead with the authors' own results and use the literature to contextualize them, not the other way around.

**Once again, we appreciate the reviewer’s in-depth consideration of our manuscript. We note this comment is slightly contradicts the opinion of reviewer 1, who wishes for the paper to have more contextualization in within the current literature. However, this reviewer raises some important areas for us to highlight further in the discussion. The confounding of ED biomarkers and mortality has been further addressed, as noted above. We have included further discussion on the a-1antitrypsin, and we have noted the reviewers interesting observation about calprotectin on. And we have added discussion regarding the high proportion of calprotectin values above what is considered the ULN for age, even at the time of discharge (L339).**

Lines 338-346: The authors conclude by recommending 'interventions to prevent re-colonization with enteropathogens' as a strategy to improve post-discharge health. However, the study did not collect any post-discharge stool samples. There are no data in this manuscript on enteropathogen burden after discharge, whether re-colonization occurred, at what rate, with which pathogens, or how it related to outcomes. The conclusion that re-colonization is the mechanism driving post-discharge mortality is entirely speculative and unsupported by the data presented. This recommendation either needs to be removed or framed as a hypothesis to be tested, not a conclusion from this study.

**This is a very fair critique, and we have reframed the statement as a hypothesis in line with the reviewer’s comment.**

***L450-453: “Collectively, these results suggest that at the point of discharge children may have relatively lower enteropathogen burden and less severe enteric inflammation and it is possible extending this period of relative enteric health may be opportunity to improve post-discharge outcomes.”***

Lines 364-373: The authors note that the association between discharge calprotectin and post-discharge mortality may reflect residual confounding by systemic inflammation. This is an important caveat but it is buried. If systemic inflammation is a plausible confounder and was not measured or adjusted for, this is a significant limitation that should be prominently stated.

**We have now moved the text to the limitations section to highlight the point made by the reviewed and we have further added to it, heightening the point raised by the reviewer:**

***L423-432: “These data were observational limiting our ability to infer causality. For example, systemic inflammation is known to be associated with post-discharge outcomes. While systemic inflammation is considered to be a downstream consequence of enteric inflammation, it is plausible that systemic inflammation may contribute to enteric inflammation. We did adjust for major diagnoses associated with systemic inflammation – namely sepsis, pneumonia, and malaria. However, systemic inflammation, especially due to other etiologies could be a residual confounder in the association between discharge calprotectin and mortality.”***

Conclusions
Lines 388-397: The conclusions overstate what the data support. The study found that discharge calprotectin was associated with 180-day post-discharge mortality after adjustment for a set of measured confounders. It did not test any intervention, did not follow children after discharge to assess re-colonization, and did not demonstrate that reducing calprotectin at discharge improves outcomes. The concluding statement that interventions to prevent enteropathogen recolonization and prolong reduced enteric inflammation 'may be useful adjuncts to discharge care' cannot be inferred from the results of this observational study. The conclusions must be restricted to what the data actually show.

**As noted in response to the similar comment above, we have revised the conclusion*s.***

Lines 388-397: cohort was oversampled for malnourished children and includes a sizeable proportion with severe wasting or kwashiorkor, HIV infection, malaria, pneumonia, and sepsis. These are not representative of all hospitalized children. The generalizability of these findings to hospitalized children without severe malnutrition or these comorbidities is entirely unclear and should be stated explicitly as a limitation.

**This is a fair comment and we have included this limitation in the revised manuscript:**

**L430-432: “Our cohort oversampled children with wasting to gain insights into patients with a high risk of mortality, but this may limit the generalizability or our findings to lower risk populations.”**

Additional Concerns:
Norovirus (Lines 229-231): The near-doubling of norovirus prevalence during hospitalization, observed consistently across seven of nine sites, is a notable signal for nosocomial acquisition. This finding has direct infection prevention and control implications. It deserves greater prominence in the discussion and should be flagged as a clinical concern, not merely noted as a descriptive finding.

**In response to this comment we have added a further statement in the paragraph of the discussion that considered our incidental finding on Norovirus.**

**L394-400: *“Norovirus prevalence increased during hospitalization across multiple sites. Norovirus acquisition was most common among children admitted with diarrhea, which may indicate missed norovirus detections at admission or that norovirus is adept at infecting children with recent diarrhea. Children with diarrhea are often accommodated in distinct units within pediatric wards which may facilitate nosocomial norovirus acquisition. This finding suggests that norovirus testing for norovirus and isolation of infected patients could prevent nosocomial spread on paediatric units in LMICs.”***

Table 2: The IQR for discharge myeloperoxidase is reported as 0.6 to 33, which appears to be a typographical error given the median is 1.4 ug/ml. This should be verified and corrected.

**This has been corrected.**

Statistical methods (Lines 166-212): P values for comparing enteropathogen prevalence across groups are described as derived from chi-square tests, but no details are provided regarding how the nested case-cohort weighting was applied to these comparisons. Given that the NCC design over-represents deaths, unadjusted proportions and chi-square tests may not reflect the true prevalence in the parent CHAIN cohort. The authors should clarify whether these prevalence estimates are weighted or unweighted.

**The reviewer makes another point about accounting for the sample selection in the prevalences given in Table. We did account for prevalences in the papers model’s but did not weight prevalence. Fortunately, because few of the pathogens and biomarkers of EED were associated with mortality the re-weighting of the sample makes very little difference to the prevalences. We have adjusted the table to be weighted for sample selection.**
