# Supplementary material for: Enteric dysfunction and enteropathogens among hospitalized south Asian and sub-Saharan African children
Source: PLOS Glob Public Health. 2026 Jul 24;6(7):e0006873. doi: 10.1371/journal.pgph.0006873 (PMC13399345; doi:10.1371/journal.pgph.0006873)
Supplement: Peer review history

# Author comment: Author response to Decision Letter 0

**DOI:** 10.1371/journal.pgph.0006873.r001

**Transfer Alert**

This paper was transferred from another journal. As a result, its full editorial history (including decision letters, peer reviews and author responses) may not be present.

14 Nov 2025

Attachment Submitted filename: Response letter_12Nov2025.pdf

# Peer review: Decision Letter 0

**DOI:** 10.1371/journal.pgph.0006873.r002

**Authors:** Ayse Ercumen

9 Mar 2026

PGPH-D-25-03032

Enteric dysfunction and enteropathogens among hospitalized south Asian and sub-Saharan African children

PLOS Global Public Health

Dear Dr. Tickell,

Thank you for submitting your manuscript to PLOS Global Public Health. After careful consideration, we feel that it has merit but does not fully meet PLOS Global Public Health’s publication criteria as it currently stands. Therefore, we invite you to submit a revised version of the manuscript that addresses the points raised during the review process.

For example, the reviewers expressed concern about elements of the analytical approach (e.g. lack of adjustment for multiple hypothesis testing, not considering the length of hospital stay, long follow-up period). Reviewers also felt that the paper would be strengthened by providing justification for some of the analytical choices, aligning the conclusions with the strength of the evidence from the study and providing broader coverage of prior evidence.

Please submit your revised manuscript by Apr 23 2026 11:59PM. If you will need more time than this to complete your revisions, please reply to this message or contact the journal office at globalpubhealth@plos.org. When you're ready to submit your revision, log on to https://www.editorialmanager.com/pgph/ and select the 'Submissions Needing Revision' folder to locate your manuscript file.

Please include the following items when submitting your revised manuscript:

- A letter that responds to each point raised by the editor and reviewer(s). You should upload this letter as a separate file labeled 'Response to Reviewers'.
- A marked-up copy of your manuscript that highlights changes made to the original version. You should upload this as a separate file labeled 'Revised Manuscript with Track Changes'.
- An unmarked version of your revised paper without tracked changes. You should upload this as a separate file labeled 'Manuscript'.

Guidelines for resubmitting your figure files are available below the reviewer comments at the end of this letter.

We look forward to receiving your revised manuscript.

Kind regards,

Ayse Ercumen, Ph.D.

Academic Editor

PLOS Global Public Health

**Journal Requirements:**

1. Please include a complete copy of PLOS’ questionnaire on inclusivity in global research in your revised manuscript. Our policy for research in this area aims to improve transparency in the reporting of research performed outside of researchers’ own country or community. The policy applies to researchers who have travelled to a different country to conduct research, research with Indigenous populations or their lands, and research on cultural artefacts. The questionnaire can also be requested at the journal’s discretion for any other submissions, even if these conditions are not met.  Please find more information on the policy and a link to download a blank copy of the questionnaire here: https://journals.plos.org/globalpublichealth/s/best-practices-in-research-reporting. Please upload a completed version of your questionnaire as Supporting Information when you resubmit your manuscript.

2. Please amend your detailed Financial Disclosure statement. This is published with the article. It must therefore be completed in full sentences and contain the exact wording you wish to be published.

i. Please clarify all sources of financial support for your study. List the grants, grant numbers, and organizations that funded your study, including funding received from your institution. Please note that suppliers of material support, including research materials, should be recognized in the Acknowledgements section rather than in the Financial Disclosure.

ii. State the initials, alongside each funding source, of each author to receive each grant. For example: "This work was supported by the National Institutes of Health (####### to AM; ###### to CJ) and the National Science Foundation (###### to AM)."

iii. State what role the funders took in the study. If the funders had no role in your study, please state: “The funders had no role in study design, data collection and analysis, decision to publish, or preparation of the manuscript.”

iv. If any authors received a salary from any of your funders, please state which authors and which funders.

3. Please upload separate figure files in .tif or .eps format. Also, remove the figures from your manuscript file but keep the legends.

For more information about figure files please see our guidelines:

https://journals.plos.org/globalpublichealth/s/figures

https://journals.plos.org/globalpublichealth/s/figures#loc-file-requirements

4. We have noticed that you have uploaded Supporting Information files, but you have not included a list of legends. Please add a full list of legends for your Supporting Information files after the references list.

If the reviewer comments include a recommendation to cite specific previously published works, please review and evaluate these publications to determine whether they are relevant and should be cited. There is no requirement to cite these works unless the editor has indicated otherwise.

**Additional Editor Comments (if provided):**

[Note: HTML markup is below. Please do not edit.]

Reviewers' comments:

Reviewer's Responses to Questions

**Comments to the Author**

1. Does this manuscript meet PLOS Global Public Health’s publication criteria? Is the manuscript technically sound, and do the data support the conclusions? The manuscript must describe methodologically and ethically rigorous research with conclusions that are appropriately drawn based on the data presented.

Reviewer #1: Yes

Reviewer #2: Partly

**********

2. Has the statistical analysis been performed appropriately and rigorously?

Reviewer #1: Yes

Reviewer #2: Yes

**********

3. Have the authors made all data underlying the findings in their manuscript fully available (please refer to the Data Availability Statement at the start of the manuscript PDF file)?

The PLOS Data policy requires authors to make all data underlying the findings described in their manuscript fully available without restriction, with rare exception. The data should be provided as part of the manuscript or its supporting information, or deposited to a public repository. For example, in addition to summary statistics, the data points behind means, medians and variance measures should be available. If there are restrictions on publicly sharing data—e.g. participant privacy or use of data from a third party—those must be specified.

Reviewer #1: Yes

Reviewer #2: Yes

**********

4. Is the manuscript presented in an intelligible fashion and written in standard English?

PLOS Global Public Health does not copyedit accepted manuscripts, so the language in submitted articles must be clear, correct, and unambiguous. Any typographical or grammatical errors should be corrected at revision, so please note any specific errors here.

Reviewer #1: Yes

Reviewer #2: Yes

**********

5. Review Comments to the Author

Please use the space provided to explain your answers to the questions above. You may also include additional comments for the author, including concerns about dual publication, research ethics, or publication ethics. (Please upload your review as an attachment if it exceeds 20,000 characters)

**Reviewer #1** : Overall Comments:

● What are the noteworthy results?

The noteworthy results of this paper are that ED biomarkers were higher in hospitalized children at admission than in community children, and that by the time hospitalized children were discharged, their ED biomarkers were lower, sometimes even lower than community children. Additionally, this paper found that ED biomarkers were higher in children who had an invasive enteropathogen detected from a rectal swab. Finally, fecal calprotectin at discharge was associated with mortality.

● Will the work be of significance to the field and related fields? How does it compare to the established literature? If the work is not original, please provide relevant references.

This study strengthens the existing evidence for a potential connection between enteric dysfunction, enteric pathogens, and mortality in hospitalized children, finding consistently that sicker children have higher ED biomarkers and more enteric pathogens, that these markers of illness improve during their hospital stay, and that discharge levels of calprotectin were associated with mortality. This study improves upon the existing literature with a larger and more diverse cohort of children.

● Does the work support the conclusions and claims, or is additional evidence needed?

Yes, the work supports the conclusions and claims.

● Are there any flaws in the data analysis, interpretation and conclusions? Do these prohibit publication or require revision?

Yes, the statistical analysis lacks adjustment for multiple comparisons. Given the magnitude of comparisons made, this should be addressed. At the very least, the choice not to make such adjustments must be defended in the text.

● Is the methodology sound? Does the work meet the expected standards in your field?

Besides the lack of adjustment for multiple comparisons, the methodology is sound.

● Is there enough detail provided in the methods for the work to be reproduced?

Yes

Major Comments:

Introduction

Page 4, Line 80: Additional context regarding the causes of post-discharge mortality would be useful. I.e. manner of death, associated dehydration, diarrhea, cardiovascular problems etc.

Page 4, Line 84: A more nuanced treatment of the interrelated conditions listed here (EED, ED, malnutrition, GI infections, systemic inflammation, and microbiome dysbiosis) is warranted. Additionally, this sentence lacks proper citation or rationale.

Page 4, Line 92-97: A more thorough review of other studies examining ED biomarkers in hospitalized children in LMICs is warranted here. There are a few I do not see cited in the paper, particularly Sturgeon 2024 and the recent Allen 2025 in Nature Comms. Going over their findings and highlighting potential differences in the authors’ study design would be a valuable addition. As this study is described in lines 94-97, be sure to highlight the timing of biomarker measurement (admission vs. discharge) and comparisons with community children.

Methods: Identification and adjustment of confounders was unclear in this section, though tables were helpful; please clarify and emphasize this part of the analysis. Additionally, please provide the coefficient of variation for the ELISA assays.

Discussion

Page 14, Line 327-329: I certainly would understand if the authors choose to address this in another work, but I would be very curious to see what additional analyses of the relationship between antibiotic use, ED biomarkers, and enteropathogen presence would reveal. For this paper, I wonder whether antibiotic use should have been run in the ED biomarker correlate analysis or as a potential confounder in every multivariate analysis performed. It would be interesting to see whether antibiotics would be associated with more detection of norovirus, for example.

Page 14, Line 329–331: Including some additional information and citations regarding the impacts of sanitation changes on ED biomarkers and enteropathogens would strengthen this hypothesis.

Page 15, Line 338-341: This idea would be strengthened if the authors would add information about the details of post-discharge morbidity and mortality here or elsewhere in the introduction/discussion.

Page 15, Line 346: Additional information about budesonide, as the authors have provided about teduglutide and mesalazine, is warranted.

Table 1: Would be valuable to add reason for hospitalization to the characteristics table, or somewhere else if more appropriate, assuming this would be different than Diagnoses. If diagnoses is equivalent to reason for hospitalization please clarify this.

Figure 2: Resolution is poor on this image. Include n for each group. Note that most papers use ng/mL to report MPO concentrations (see Kosek 2013). Keeping units the same from paper to paper is important for the purpose of comparison. It also appears the authors use different units in tables vs. this figure, please amend. This image tells a strong story that did not come off so clearly in the text of the results - perhaps consider strengthening the text for this section.

S2 Table 6: This table should be included in the main text given that it is a whole section of the results.

Minor Comments:

Page 3, Line 65: strike “was associated with mortality”; redundant with first part of sentence.

Page 4, Line 82: add microbial to specify what kind of translocation

Page 4, Line 92: would be valuable to add some additional citations after enteroinvasive to further support the consensus on the relationship between ED biomarkers and enteropathogens.

Page 10, Line 229: Awk sentence, recommend adding a semicolon after “During hospitalization norovirus presences nearly doubled”

Page 14, Lines 313-316: Recommend reordering these two sentences so that the statement outlining how the authors’ evidence supports the hypothesis concludes the paragraph.

Page 14, Line 325: typo; change “his” to “this”

Page 15, Line 349: Formatting may have been altered by forces outside the authors’ control but be sure to italize latin name for P. falciparum malaria.

Page 17, Line 381: typo; change “diagnostic is” to “diagnostics are”

Table 3: Appears to be a missed bold or typo in None vs. exclusive breastfeeding; a-1-antitrypsin for adjusted comparison; Coef is positive, 95% CI is negative. Typo in second line under table. Change “due” to “of”. Appears to be minor alignment issue with center column as coef’s are lower than corresponding CIs.

**Reviewer #2** : This manuscript addresses an important and underexplored question regarding enteric dysfunction and enteropathogen burden in hospitalized children across LMICs. The data are drawn from the well-established CHAIN cohort and benefit from a large, multi-site, multi-country design. However, the manuscript has several substantive methodological, analytical, and interpretive concerns that must be addressed before it can be considered for publication. The specific issues are detailed below, organized by section, with line numbers provided for the authors' reference.

Abstract

Lines 48-49 vs. Lines 199-200: abstract states that cox-proportional hazard models assessed ED biomarker associations with 'inpatient and post-discharge mortality.' while methods later clarifies that 30-day period following admission was chosen. Why are these two framings contradictory? The abstract must be corrected to accurately reflect what was actually measured.

Lines 53-55: Calprotectin at discharge was significantly lower than community, yet the abstract goes on to report that discharge calprotectin was the only biomarker associated with mortality. If discharge calprotectin was not elevated, the premise that enteric inflammation at admission is a clinically important finding in this hospitalized group becomes difficult to defend. The authors need to address why a biomarker that behaves no differently from the community would carry mortality-predictive value at discharge.

Introduction

Lines 78-97: The introduction is well-structured and provides limited background. However, it does not provide the specific hypothesis being tested regarding whether enteric dysfunction in acutely ill hospitalized children is etiologically distinct from environmental enteric dysfunction in community children. This distinction is critical for interpreting the results and should be stated more explicitly.

Methods

Lines 128-132: What is the justification for nested case-cohort (NCC) randomly selected 24% of children from the CHAIN cohort along with all deaths. Was a formal sample size calculation performed to ensure this sub-cohort was powered to detect associations between ED biomarkers and mortality? If so, the assumed effect sizes, alpha, and power should be reported. If not, this is a significant methodological gap that must be acknowledged.

Lines 129-132: The decision to recruit 30 community children per site also lacks justification.

Lines 199-201: The authors state that the 30-day period following hospital admission was chosen because 'inpatient mortality is biased by discharge decisions.' While this reasoning is understandable, labelling this as inpatient mortality anywhere in the manuscript is inaccurate. Children discharged within that 30-day window who subsequently die are not inpatient deaths.

Lines 199-204: Using ED biomarkers measured at admission to predict mortality over the subsequent 30 days, without accounting for or even reporting the number of days each child remained hospitalized, is a fundamental flaw. A child admitted with a high myeloperoxidase value who dies on day two of admission versus from one who survives, is treated, and dies on day 28? The length of hospital stay is not reported anywhere in the manuscript, no mean or median duration of admission is provided despite the discussion (line 327) mentioning 'an average of five days.' If this figure exists, it belongs in Table 1 or the results section, and its implications for the mortality models must be examined.

Lines 206-211: Using discharge biomarker concentrations to predict mortality over 180 days following discharge is a very long causal window. The biological rationale for why a stool biomarker measured at the point of discharge would independently predict mortality six months later, against the backdrop of all the life events, re-infections, nutritional deterioration, or new illnesses that could occur in that period, is not adequately established. The authors must provide a scientific justification for this association as it is highlighted in the conclusions that is drawn from it.

Lines 202-210: MUAC is used as a covariate in both the admission and discharge models. The authors should clarify whether admission MUAC or discharge MUAC is used in each respective model. More importantly, it is biologically implausible that MUAC would change meaningfully over a median hospital stay of five days in these children. If admission MUAC is used in the discharge model, this needs to be justified. If discharge MUAC is used, the authors need to explain what meaningful change is expected in this short a window in acutely ill malnourished children, and whether this creates collinearity with admission MUAC.

Results

Line 218: The statement that hospitalized children were 'less commonly exclusive breastfed' requires clarification. A comparison of breastfeeding rates across the broad age group confound the age-appropriate feeding practices with what may simply be an age distribution difference between the hospitalized and community groups. This should be stratified by age group or the comparison should be appropriately caveated.

Lines 216-222: Table 1 provides sociodemographic and clinical characteristics but does not report the distribution of MUAC strata (no wasting, moderate wasting, severe wasting or kwashiorkor) separately for the hospitalized group. Given that enrollment was deliberately stratified by MUAC in a 2-1-2 ratio, and that MUAC is strongly associated with both biomarker levels and mortality, the reader needs to know how many children fell into each nutritional stratum. Failure to present this renders the clinical context of the acutely ill cohort incomplete and makes it impossible to assess the degree of confounding by nutritional status.

Lines 225-231: The paper compares the prevalence of dehydrating pathogens between hospitalized and community children and reports a significant difference (25-27% vs 8%, p<0.001). While this is numerically striking, the clinical relevance of this comparison is questionable. Children admitted to hospital are there precisely because they are acutely unwell, and a significant proportion presented with diarrhea. Comparing pathogen prevalence for dehydrating organisms in acutely ill hospitalized children with healthy community children tells us little beyond the expected. The authors should clarify what clinical or public health inference they intend the reader to draw from this comparison.

Lines 242-243: Giardia was lower among hospitalized children at both admission and discharge compared to community children (7% vs 11%), and this finding is never discussed. Given that Giardia is associated with asymptomatic carriage and positive growth outcomes in some cohorts, its higher prevalence in the community group warrants at least a brief acknowledgment in the discussion.

Lines 246-252: The alpha-1-antitrypsin findings are counterintuitive and insufficiently explained in the results. Concentrations are lower at admission compared to the community, but then recover to community levels by discharge. The direction of this change is opposite to that of the inflammatory markers and the biological interpretation is unclear. The authors invoke a dilutional effect of diarrhea in the discussion, but this is not examined rigorously in the results. Given that this biomarker reflects intestinal permeability, not inflammation, and given that these children are acutely ill with multiple conditions that could compromise gut barrier function, this unexpected pattern deserves a proper mechanistic discussion rather than a brief mentioning.

Lines 254-256: The text states that 'trends in pathogen prevalence were similar among hospitalized children with and without reported diarrhea,' yet the very next sentence reports that children with diarrhea had higher rotavirus, norovirus, typical EPEC, and Shigella prevalence. These two statements are not consistent. If the distributions of individual pathogens differ substantially by diarrhea status, the overall trends cannot be described as similar without qualification.

Lines 267-285 (Table 3): The correlate models in Table 3 are restricted to the hospitalized cohort. No equivalent model is presented for community children. It is therefore unknown whether the association between lower MUAC and higher myeloperoxidase or calprotectin is specific to the acutely ill or is equally present in the community. The same applies to the association with invasive enteropathogens. Without a community reference model, it is impossible to determine whether these associations are a feature of acute illness or simply reflect the biology of these children in their home environment.

Lines 267-285 (Table 3): Acutely ill children in this cohort have a high prevalence of sepsis (13.4%), pneumonia (39.6%), and malaria (12.6%). Fecal biomarkers such as myeloperoxidase and calprotectin reflect systemic as well as local intestinal inflammation. In children with these co-morbidities, attributing elevated biomarker values to enteropathogen detection, whether or not diarrhea is present, is methodologically problematic. The presence of these systemic illnesses is a major unmeasured confounder for the enteropathogen-biomarker relationship, and this is insufficiently acknowledged.

Lines 293-297: The crude models found that admission myeloperoxidase and calprotectin were associated with 30-day mortality, but this association was lost after adjustment. This is an important null finding that deserves more interpretive weight in the discussion. If the biomarker-mortality relationship at admission disappears entirely after adjusting for MUAC, age, and co-morbidities, then the biomarkers add no independent prognostic information at admission. The authors should reflect on what this means for the utility of measuring these biomarkers at admission?

Lines 299-304: The finding that discharge calprotectin is associated with 180-day post-discharge mortality, while no admission biomarker retains significance after adjustment, raises the question of whether this reflects a true biological relationship or residual confounding. Discharge calprotectin was already lower than community levels (171.2 vs 252.4 ug/ml). Explaining how a biomarker that is already below community norms at discharge independently predicts mortality six months later requires more than a hazard ratio; it requires a mechanistic and contextual argument that is not currently present.

Lines 299-304: The text does not clearly specify for which model admission versus discharge biomarker values were used. The description of crude and adjusted models for 30-day and 180-day outcomes would benefit from explicit clarification in each case as to which timepoint's biomarker was entered into each model.

Lines 299-304: The HIV-infected rate in this cohort is 6.7%. HIV infection is independently associated with enteropathy, impaired immunity, and mortality. The handling of HIV-infected children in the mortality models is not described. Were they excluded, included without stratification, or included with HIV status as a covariate? Given the known impact of HIV on both enteric inflammation and post-discharge mortality, this requires explicit reporting.

Figure 2

Figure 2: A dedicated figure is presented comparing myeloperoxidase at admission and discharge, stratified by invasive pathogen detection. However, myeloperoxidase was not associated with mortality in either crude or adjusted post-discharge models. Presenting a detailed figure for a biomarker that does not ultimately predict the primary outcome of interest, while calprotectin, which did retain significance, is not similarly visualized, represents a disconnect between the analytical focus and the visual emphasis of the manuscript. The authors should reconsider this figure or add a parallel figure for calprotectin.

Discussion

Lines 307-397: The discussion section reads largely as an overview of the broader literature on enteric dysfunction and EED rather than an interpretation of the authors' own findings. Key results from this study, particularly the null findings for myeloperoxidase and alpha-1-antitrypsin in the mortality models, the counterintuitive direction of alpha-1-antitrypsin across timepoints, and the paradox of below-community calprotectin at discharge predicting mortality six months later, are not given adequate analytical attention. The discussion should be restructured to lead with the authors' own results and use the literature to contextualize them, not the other way around.

Lines 338-346: The authors conclude by recommending 'interventions to prevent re-colonization with enteropathogens' as a strategy to improve post-discharge health. However, the study did not collect any post-discharge stool samples. There are no data in this manuscript on enteropathogen burden after discharge, whether re-colonization occurred, at what rate, with which pathogens, or how it related to outcomes. The conclusion that re-colonization is the mechanism driving post-discharge mortality is entirely speculative and unsupported by the data presented. This recommendation either needs to be removed or framed as a hypothesis to be tested, not a conclusion from this study.

Lines 364-373: The authors note that the association between discharge calprotectin and post-discharge mortality may reflect residual confounding by systemic inflammation. This is an important caveat but it is buried. If systemic inflammation is a plausible confounder and was not measured or adjusted for, this is a significant limitation that should be prominently stated.

Conclusions

Lines 388-397: The conclusions overstate what the data support. The study found that discharge calprotectin was associated with 180-day post-discharge mortality after adjustment for a set of measured confounders. It did not test any intervention, did not follow children after discharge to assess re-colonization, and did not demonstrate that reducing calprotectin at discharge improves outcomes. The concluding statement that interventions to prevent enteropathogen recolonization and prolong reduced enteric inflammation 'may be useful adjuncts to discharge care' cannot be inferred from the results of this observational study. The conclusions must be restricted to what the data actually show.

Lines 388-397: cohort was oversampled for malnourished children and includes a sizeable proportion with severe wasting or kwashiorkor, HIV infection, malaria, pneumonia, and sepsis. These are not representative of all hospitalized children. The generalizability of these findings to hospitalized children without severe malnutrition or these comorbidities is entirely unclear and should be stated explicitly as a limitation.

Additional Concerns:

Norovirus (Lines 229-231): The near-doubling of norovirus prevalence during hospitalization, observed consistently across seven of nine sites, is a notable signal for nosocomial acquisition. This finding has direct infection prevention and control implications. It deserves greater prominence in the discussion and should be flagged as a clinical concern, not merely noted as a descriptive finding.

Table 2: The IQR for discharge myeloperoxidase is reported as 0.6 to 33, which appears to be a typographical error given the median is 1.4 ug/ml. This should be verified and corrected.

Statistical methods (Lines 166-212): P values for comparing enteropathogen prevalence across groups are described as derived from chi-square tests, but no details are provided regarding how the nested case-cohort weighting was applied to these comparisons. Given that the NCC design over-represents deaths, unadjusted proportions and chi-square tests may not reflect the true prevalence in the parent CHAIN cohort. The authors should clarify whether these prevalence estimates are weighted or unweighted.

Major revision required. The manuscript addresses a clinically relevant question using a well-designed multi-site cohort, but the current version has major gaps in the reporting of basic descriptive statistics, an insufficient scientific basis for several key analytical choices, an overclaiming of conclusions relative to the data, and a discussion that does not adequately support the results or defend the findings. The concerns raised above must be addressed systematically before this work is suitable for publication.

**********

6. PLOS authors have the option to publish the peer review history of their article (what does this mean?). If published, this will include your full peer review and any attached files.

**Do you want your identity to be public for this peer review?**  If you choose “no”, your identity will remain anonymous but your review may still be made public.

For information about this choice, including consent withdrawal, please see our Privacy Policy.

Reviewer #1: No

Reviewer #2: **Yes:** Zehra Jamil

**********

[NOTE: If reviewer comments were submitted as an attachment file, they will be attached to this email and accessible via the submission site. Please log into your account, locate the manuscript record, and check for the action link "View Attachments". If this link does not appear, there are no attachment files.]

-->Figure Resubmissions:

While revising your submission, we strongly recommend that you use PLOS’s NAAS tool (https://ngplosjournals.pagemajik.ai/artanalysis) to test your figure files. NAAS can convert your figure files to the TIFF file type and meet basic requirements (such as print size, resolution), or provide you with a report on issues that do not meet our requirements and that NAAS cannot fix.

After uploading your figures to PLOS’s NAAS tool - https://ngplosjournals.pagemajik.ai/artanalysis, NAAS will process the files provided and display the results in the "Uploaded Files" section of the page as the processing is complete. If the uploaded figures meet our requirements (or NAAS is able to fix the files to meet our requirements), the figure will be marked as "fixed" above. If NAAS is unable to fix the files, a red "failed" label will appear above. When NAAS has confirmed that the figure files meet our requirements, please download the file via the download option, and include these NAAS processed figure files when submitting your revised manuscript.

Attachment Submitted filename: Review.pdf

# Author comment: Author response to Decision Letter 1

**DOI:** 10.1371/journal.pgph.0006873.r003

12 May 2026

Attachment Submitted filename: Reviewer_comment_response_05May_2026.docx

# Peer review: Decision Letter 1

**DOI:** 10.1371/journal.pgph.0006873.r004

**Authors:** Ayse Ercumen

30 Jun 2026

Enteric dysfunction and enteropathogens among hospitalized south Asian and sub-Saharan African children

PGPH-D-25-03032R1

Dear Dr. Tickell,

We are pleased to inform you that your manuscript 'Enteric dysfunction and enteropathogens among hospitalized south Asian and sub-Saharan African children' has been provisionally accepted for publication in PLOS Global Public Health.

Before your manuscript can be formally accepted you will need to complete some formatting changes, which you will receive in a follow up email. A member of our team will be in touch with a set of requests.

Please note that your manuscript will not be scheduled for publication until you have made the required changes, so a swift response is appreciated.

IMPORTANT: The editorial review process is now complete. PLOS will only permit corrections to spelling, formatting or significant scientific errors from this point onwards. Requests for major changes, or any which affect the scientific understanding of your work, will cause delays to the publication date of your manuscript.

If your institution or institutions have a press office, please notify them about your upcoming paper to help maximize its impact. If they'll be preparing press materials, please inform our press team as soon as possible -- no later than 48 hours after receiving the formal acceptance. Your manuscript will remain under strict press embargo until 2 pm Eastern Time on the date of publication. For more information, please contact globalpubhealth@plos.org.

Thank you again for supporting Open Access publishing; we are looking forward to publishing your work in PLOS Global Public Health.

Best regards,

Ayse Ercumen, Ph.D.

Academic Editor

PLOS Global Public Health

***********************************************************

Reviewer Comments (if any, and for reference):

Reviewer's Responses to Questions

**Comments to the Author**

1. If the authors have adequately addressed your comments raised in a previous round of review and you feel that this manuscript is now acceptable for publication, you may indicate that here to bypass the “Comments to the Author” section, enter your conflict of interest statement in the “Confidential to Editor” section, and submit your "Accept" recommendation.

Reviewer #1: All comments have been addressed

**********

2. Does this manuscript meet PLOS Global Public Health’s publication criteria? Is the manuscript technically sound, and do the data support the conclusions? The manuscript must describe methodologically and ethically rigorous research with conclusions that are appropriately drawn based on the data presented.

Reviewer #1: Yes

**********

3. Has the statistical analysis been performed appropriately and rigorously?

Reviewer #1: Yes

**********

4. Have the authors made all data underlying the findings in their manuscript fully available (please refer to the Data Availability Statement at the start of the manuscript PDF file)?

The PLOS Data policy requires authors to make all data underlying the findings described in their manuscript fully available without restriction, with rare exception. The data should be provided as part of the manuscript or its supporting information, or deposited to a public repository. For example, in addition to summary statistics, the data points behind means, medians and variance measures should be available. If there are restrictions on publicly sharing data—e.g. participant privacy or use of data from a third party—those must be specified.

Reviewer #1: Yes

**********

5. Is the manuscript presented in an intelligible fashion and written in standard English?

PLOS Global Public Health does not copyedit accepted manuscripts, so the language in submitted articles must be clear, correct, and unambiguous. Any typographical or grammatical errors should be corrected at revision, so please note any specific errors here.

Reviewer #1: Yes

**********

6. Review Comments to the Author

Please use the space provided to explain your answers to the questions above. You may also include additional comments for the author, including concerns about dual publication, research ethics, or publication ethics. (Please upload your review as an attachment if it exceeds 20,000 characters)

Reviewer #1: The authors have addressed all my major concerns.

I found two dropped words that might be considered for minor revisions:

Page 4, Line 81: add "a" after "attributable to"

Page Line 453: add "an" before "opportunity"

**********

7. PLOS authors have the option to publish the peer review history of their article (what does this mean?). If published, this will include your full peer review and any attached files.

**Do you want your identity to be public for this peer review?**  If you choose “no”, your identity will remain anonymous but your review may still be made public.

For information about this choice, including consent withdrawal, please see our Privacy Policy.

Reviewer #1: No

**********
